# Multi-omics analysis of interspecies interactions in a soil *Streptomyces* community provides functional insights into siderophore ecology

**DOI:** 10.1038/s41598-026-45368-6

**Published:** 2026-04-06

**Authors:** J. A. Connolly, F. Del Carratore, K. Schmidt, A. T. Bisesi, J. N. V. Martinson, J. Chua, M. Kuhs, M. M. Boneza, S. C. Heinsch, L. L. Kinkel, M. J. Smanski, W. R. Harcombe, R. Breitling, E. Takano

**Affiliations:** 1 Manchester Institute of Biotechnology, Department of Chemistry, School of Natural Sciences, Faculty of Science and Engineering, University of Manchester, Manchester, M1 7DN UK; 2 Department of Biochemistry, Cell and Systems Biology, Institute of Integrative, Systems and Molecular Biology, University of Liverpool, Liverpool, L69 3BX UK; 3 Division of Immunology, Immunity to Infection and Respiratory Medicine, University of Manchester, Manchester, UK; 4 Department of Ecology, Evolution and Behaviour, University of Minnesota, St. Paul, MN USA; 5 Innovative Genomics Institute, University of California, Berkeley, CA USA; 6 Department of Biochemistry, Molecular Biology, and Biophysics, University of Minnesota, St. Paul, MN USA; 7 Department of Plant Pathology, University of Minnesota, St. Paul, MN USA; 8 H Biotechnology Institute, University of Minnesota, St. Paul, MN USA

**Keywords:** Soil microbiome, Transcriptomics, Metabolomics, *Streptomyces*, Desferrioxamine, Siderophore, Iron competition, CRISPR base editing, Biotechnology, Microbiology

## Abstract

*Streptomyces* are key contributors to soil microbiome function, known for their biosynthetic diversity. While advances in -omics technologies have improved our understanding of microbiome composition and metabolic potential, the mechanisms underpinning interspecies interactions remain poorly resolved. Here, we investigate the molecular basis of interactions among four sympatric *Streptomyces* soil microbiome isolates, focusing on phenotypic, metabolomic and transcriptomic responses. Co-culture experiments revealed that one isolate, strain A, exhibited pronounced phenotypic changes when grown alongside each of the other three strains. Untargeted metabolomics and RNA-seq analyses showed that strain A undergoes distinct metabolic and transcriptional shifts depending on its partner, with the strongest response elicited by strain C. Despite all four strains possessing a conserved desferrioxamine biosynthetic gene cluster, only strain C constitutively produced desferrioxamine B (DFO-B), a hydroxamate siderophore, indicating a role for iron bioavailability in the interaction. Supplementation with DFO-B or iron mimicked the growth stimulation of strain A observed in co-culture with strain C, and CRISPR base editing of *desD* in strain C abolished both DFO production and the phenotypic induction of strain A. However, transcriptomic profiles of strain A varied significantly depending on the partner strain, with distinct sets of biosynthetic gene clusters and metabolic pathways activated in response to strains B and C, suggesting additional cues beyond DFO-B. In contrast, strain D did not elicit growth stimulation in its partners, and itself showed downregulation of amino acid and carbon metabolism when co-cultured with strain C. These findings indicate that *Streptomyces* interactions are not only mediated by siderophore piracy but also involve complex, strain-specific molecular responses. Our findings demonstrate that *Streptomyces* interactions are highly strain-specific and only partly mediated by siderophore piracy, with DFO-B acting as a potent interspecies cue. The divergent molecular responses to different partners suggest nuanced mechanisms of microbial sensing and competition. These insights advance our understanding of microbial crosstalk and highlight the ecological and evolutionary complexity of siderophore-mediated interactions. By integrating transcriptomics, metabolomics, and biochemical assays, we present a robust framework for dissecting microbial interactions, with implications for microbiome engineering and synthetic community design.

**Supplementary Information:**

The online version contains supplementary material available at 10.1038/s41598-026-45368-6.

## Introduction

The diversity, composition and cooperative function of the soil microbiome are of key importance to plant health. These functions range from disease suppression to plant growth promotion^1–3^. With advances in –omics methodologies, we are better able to identify which organisms and what genes are present in microbiomes, increasingly in a spatially resolved fashion^4^. However, the effective function of the microbiome is also determined by the interspecies and even interkingdom interactions between these organisms. Understanding these interactions has in general proven more elusive.

A key bacterial genus of the soil microbiome is *Streptomyces*, which is particularly well-studied for its capacity to produce diverse secondary metabolites that have often been leveraged as high-value compounds such as antibiotics^5^. Production of such compounds is often regulated by intercellular small molecule signals like γ-butyrolactones^6^, chemical cues from neighbouring organisms^7^, and global developmental transcriptional/translational genes (*whi/bld*)^8^. Indeed, restoration of a functional *bldA* copy to allow aerial differentiation has successfully activated a ‘silent’ biosynthetic gene cluster (BGC) in *Streptomyces*^9^. In this study, we distinguish between signals and cues: signals are evolved characteristics or behaviours that convey information and confer mutual benefit to both sender and receiver, whereas cues are molecules or traits that provide information to a receiver without necessarily benefiting the emitter^10^.

*Streptomyces* are capable of synthesising molecules that drive interkingdom and interspecies interactions. For example, *Streptomyces* can utilise volatile compounds such as geosmin to recruit insects to aid the spread of their spores^11^. *Streptomyces* are also often present in insect microbiomes, where they can form mutualistic interactions such as the protection of bees from insect pathogens^12,13^. Interactions can also be mediated by antibiotics at sub-inhibitory concentrations^14,15^ or alternative natural products (NPs) such as arginoketides^16^. In laboratory settings, sympatric *Streptomyces* display antagonistic interactions^17^, yet in nature co-exist together with other diverse bacterial, archaeal, and eukaryotic taxa^18^.

An important factor that drives many soil microbiome interactions is the bioavailability of iron. Iron is both essential and toxic (at high intracellular concentrations) to all bacteria. Therefore, complex mechanisms have evolved for the precise control of iron homeostasis, and the scavenging of iron from the environment. Soil microbes, including *Streptomyces*, widely utilise secreted siderophores to scavenge iron. These are small molecules produced by microbes and plants, which tightly bind available iron, allowing for its uptake^19^. Iron bioavailability is a key determinant of microbiome competition; bacteria can use siderophore production, uptake and lack thereof, to regulate community and host interactions^20–23^. Such siderophore-mediated interactions have also been observed in *Streptomyces*^24–26^. However, not all strategies for the control of intracellular iron in *Streptomyces* are fully understood^27^.

The engineering of soil microbiomes, such as through synthetic biology, is promising for real-world applications to crops^28^. However, we must understand how these microbes interact within a community before these benefits can be realised in full in a persistent manner. *Streptomyces* species play a pivotal role in soil microbiomes, and understanding their mechanisms of interaction is particularly important, especially considering their biosynthetic talents.

**Fig. 1:**
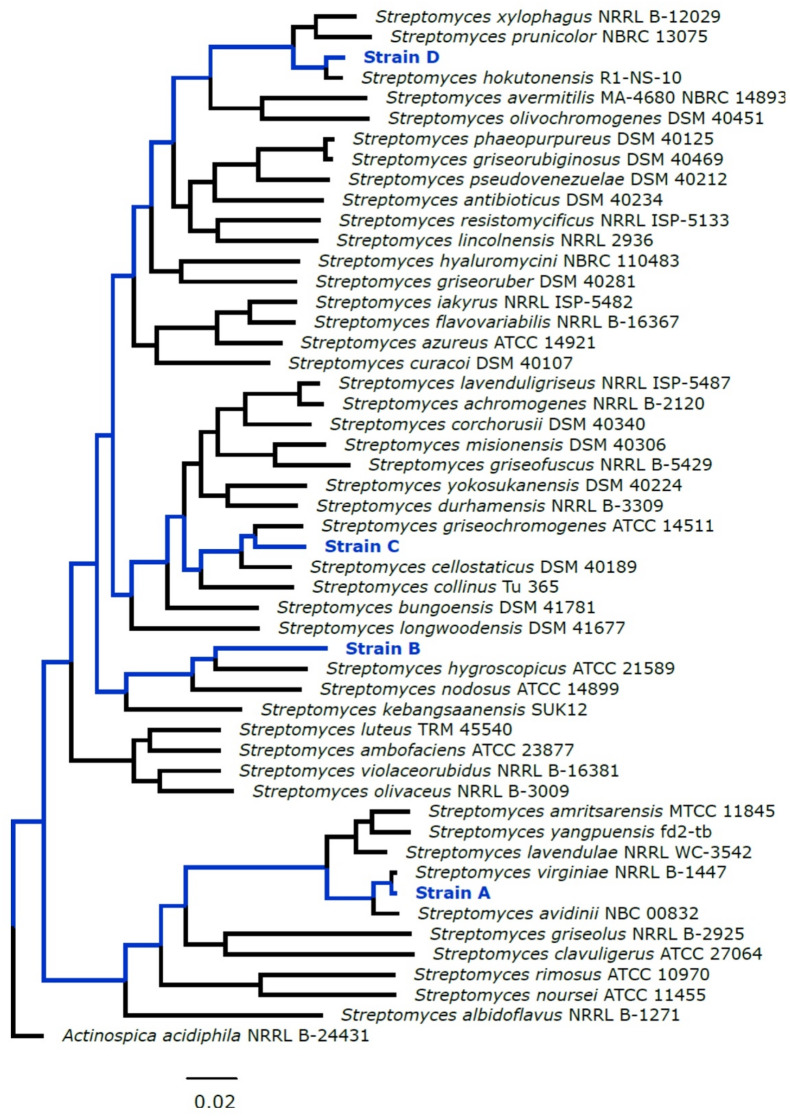
Phylogenetic tree of the four environmental isolates with selected type strains. Generated using AutoMLST^30^. The conserved genes aligned for generation of this are listed in Supplementary Table S1.

Here we explore a community of four sympatric *Streptomyces* isolates, observing their interactions and antagonistic effects phenotypically, biochemically and with untargeted metabolomics and transcriptomics. In particular, we explore a strong interaction phenotype governed by piracy of hydroxamate iron chelators. The *Streptomyces* strains investigated in this study, referred to here as strains A, B, C, D, are a subset of those isolated from a single soil core from the Cedar Creek Ecosystem Science Reserve in Minnesota, USA, as described previously^29^. ANI comparisons (Supplementary Table S2) and autoMLST^30^ phylogenetic analysis (Fig. 1) indicate that each isolate is closest to a different described *Streptomyces* species. Strains A and D meet the 95% threshold for placement as known named species, at 98.1% to *Streptomyces virginiae* and 96% to *Streptomyces hokutonensis* respectively. Strains B and C do not meet this threshold to any named species. These isolates were previously observed to participate in pairwise inhibitory interaction networks with other sympatric *Streptomyces*, displaying a high degree of antagonism^17^.

## Results

### Environmental *Streptomyces* show distinctive phenotypic and inhibitory interactions

**Fig. 2:**
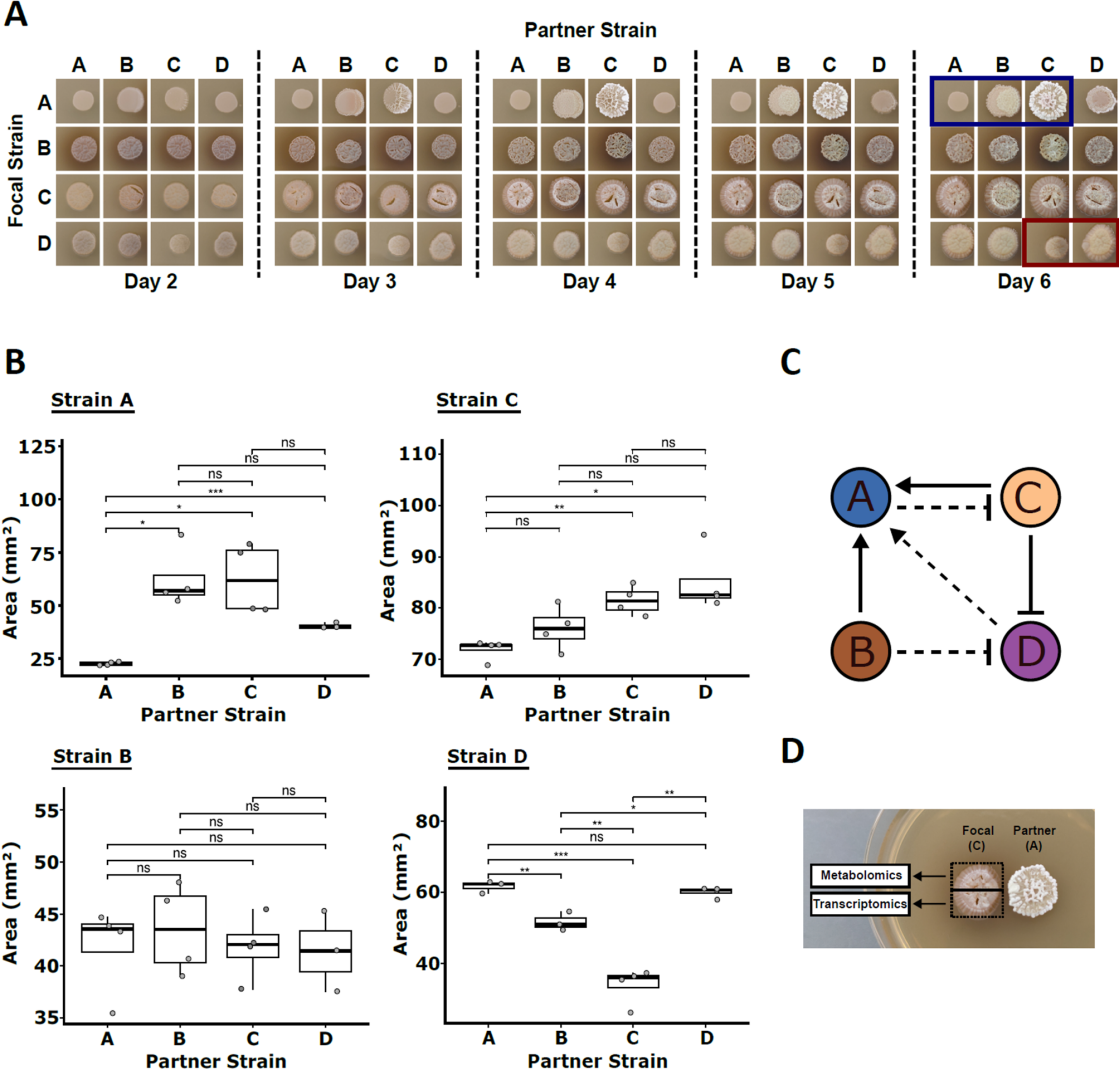
(**A**) Daily interaction phenotypes on ISP2 agar, *n* = 3. Rows represent the focal strain photographed. The partner strain (indicated by the column) is always to the right of the photographed focal strain in these images. The red and blue outlines highlight major phenotypic changes in the focal strain in response to partners. Close-up images of the interaction between A and C are included in Figure S14. Where the focal strain and partner strain are the same, these are grown next to each other in the same manner as when the partner is different. (**B**) Box plots of algorithmically determined growth areas on day 6, *n* = 4. Significance levels are indicated as follows: *** = *P* < 0.001, ** = *P* < 0.01, * = *P* < 0.05, ns = not significant. (**C**) Strain interaction network summary. Arrows represent growth promotion, bars represent inhibition/competition, dashes represent weaker effects. (**D**) Interaction phenotype analysis: strains were inoculated 1 cm apart and sampled daily from day 2 to 6 in triplicate, with half the spot used for metabolomic and transcriptomic sample preparation as indicated.

The environmental *Streptomyces* strains were inoculated in pairs 1 cm apart, and phenotypes were monitored daily from 2 to 6 days post-inoculation (Fig. 2A). We observed distinct phenotypic interactions, the most striking was the positive effect of strain C on the colony area of strain A. In the experimental conditions, when grown axenically, strain A fails to increase in colony area after day 2. Yet it exhibits clear directional growth towards partner strains, most markedly to strain C, with strain A growing to a larger surface area, accompanied by formation of aerial hyphae as indicated by the white colour of the macrocolony. A similar response is observed when A is paired with B and D, to lessening degrees. Automated measurement of growth areas on day 6 confirmed that strain A responds significantly to all partner strains when compared with itself (Fig. 2B). Conversely, with its growth unleashed, strain A inhibits or outcompetes the growth of strain C, with a visible directional interface (Fig. 2A, blue outline); growth in strain C was significantly (*P* < 0.01) decreased when next to A compared with itself on day 6 (Fig. 2B). Presence of strain C inhibits the growth of strain D in a non-directional manner (Figs. 2A, red outline; Fig. 2B; Figure S14). The paired growth interaction phenotypes are summarised in Fig. 2C. Transcriptomic and metabolomics approaches were undertaken to investigate the molecular drivers of these interactions, and the effects they have on receiving strains.

### Untargeted metabolomics and transcriptomics indicate extensive responses to partner strains

As shown in Fig. 2D, samples for untargeted metabolomics and transcriptomics were harvested from each interaction plate. For the metabolomics analysis, rectangular plugs containing both agar and mycelia were excised from the plate in triplicate, on days 2 through 6. Cold methanol extractions of these were analysed on a Q Exactive Plus MS instrument after UHPLC separation on a C18 column. Peaks were identified using mzMatch^31^, and annotation was performed using the integrated probabilistic annotation (IPA) method^32^^33^. The effect of the pre-processing pipeline is shown in Supplementary Figure S7.

**Fig. 3:**
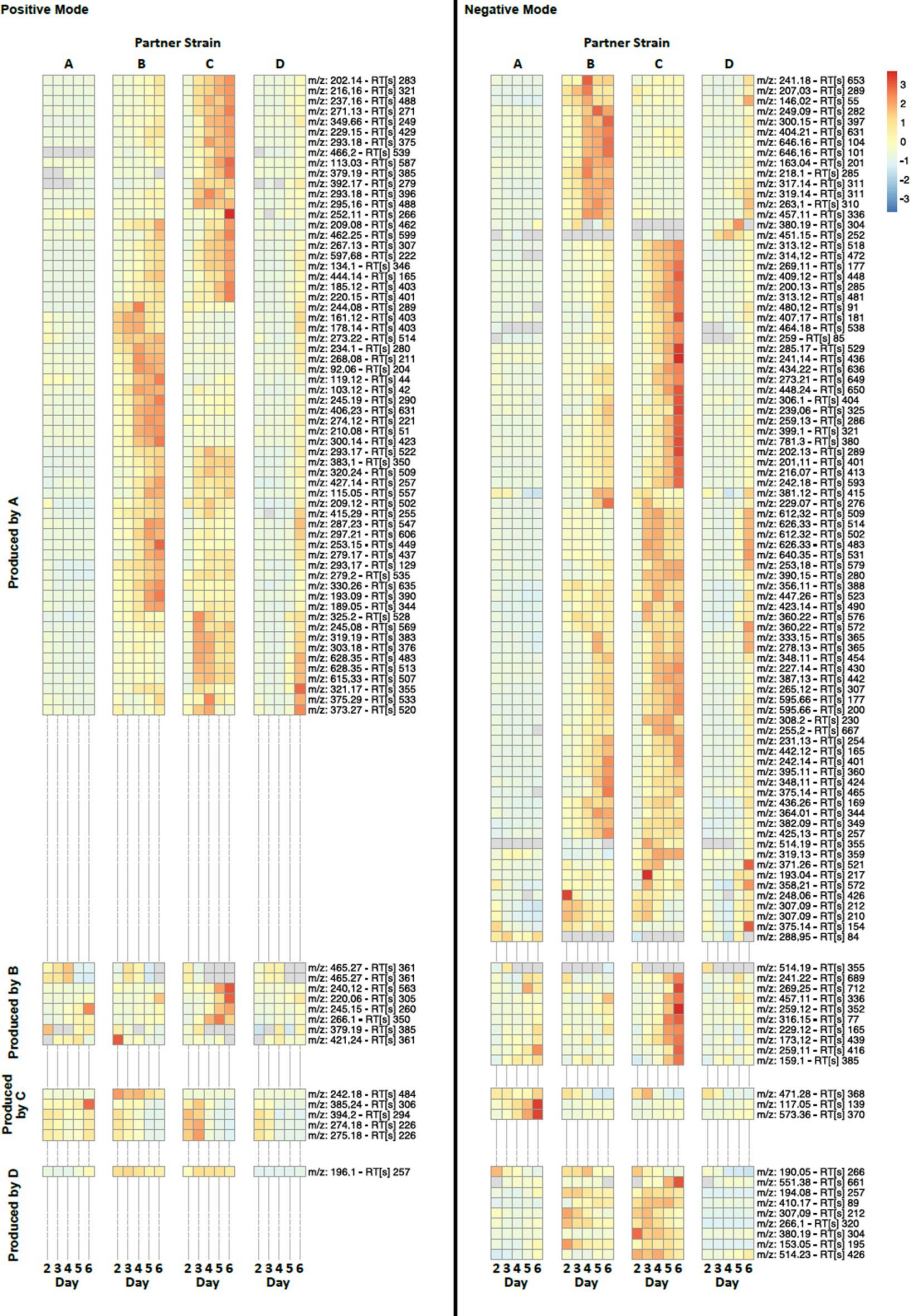
Heatmaps of the normalised intensities associated with the ions likely produced by the different strains. The peaks to display were chosen based on significant difference from self (e.g. A-C vs. A-A) and the inverse partner (e.g. A-C vs. C-A) in at least one time point. Intensities were row-scaled (Z-scores) to visualise relative intensity patterns. Ions detected in positive mode are on the left and in negative mode on the right. Each column represents one sampling day, and each group of columns represents one partner strain.

**Fig. 4:**
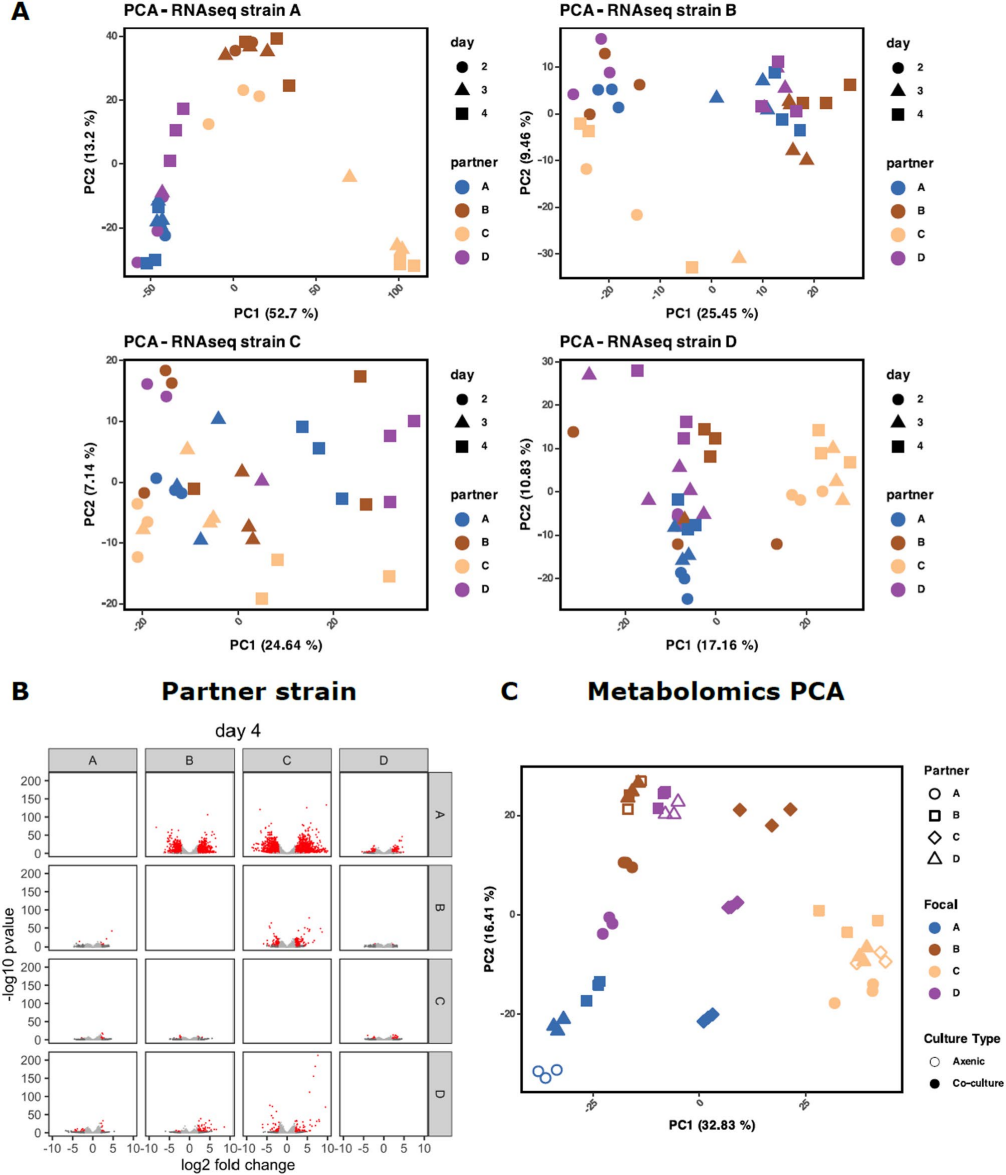
(**A**) Temporal PCA of transcriptomics datasets. (**B**) Volcano plots of fold change with respect to axenic pairings on day 4. Genes with significant adjusted p-values (< 0.05) and a log_2_fc > 2 or < − 2 are indicated in red. (**C**) PCA of detected LC-MS peaks on day 4. Intensity data for peaks collected in negative and positive modes were cleaned and aggregated.

The analysis detected 7187 main peaks in positive mode and 3539 in negative mode. The PCA score plot generated from the metabolomics data acquired in positive mode for the day 4 samples is shown in Fig. 4C. To identify the ions associated with the metabolites produced by each strain in response to different partners, we performed the statistical analysis described in the Methods section. Briefly, when looking for peaks produced by A in response to C, we selected peaks showing a strong statistically significant increase (*p*_*adj*_ < = 0.05 and log2FC > = 1.5) in at least one time point in each of the following two comparisons: A-C vs. A-A and A-C vs. C-A. The results of such analysis are summarized in the heatmaps shown in Fig. 3. All features selected for this analysis are shown in all comparisons, irrespective of the comparison they were selected in. The majority of such filtered ions were not matched to the IPA reference databases, or produced matches not known to be produced by *Streptomyces* (Supplementary Figure S6). This could be because such interaction-emergent compounds from non-model *Streptomyces* isolates are more likely to be uncommon or unknown.

Consistent with the phenotypic response (Fig. 2A), most peaks selected by this analysis are produced by strain A (Fig. 3). It is worth noting that strain A shows a different metabolic signature depending on which strain it is growing next to. It is particularly interesting to notice that metabolites seemingly produced by strain A in response to strain B are not produced in response to strain C (and vice versa), suggestive of a strain-specific metabolic response by strain A.

From the same interaction plates, samples for transcriptomics analysis were also collected. To explore the transcriptional landscape of our interacting strains, RNA-seq was performed in triplicate on days 2, 3 and 4. Differential analysis with DEseq2^34^ revealed a strong correlation between transcriptional response and phenotype (Figs. 2A and 4A and B). When growing next to itself or to strain D, strain A has little increase in size after day 2. Conversely, strain A growth greatly increases when next to strain B and strain C which likely explains the high number of differentially expressed genes identified. Summaries of the response of BGC core gene expression are included in Supplementary Figures S1-S4.

Enrichment analysis was performed to determine if pathways, as determined by KEGG^35,36^,^37^ annotation, were significantly overrepresented as differentially regulated in the RNA-seq dataset. Despite extensive transcriptional shifts in strain A, no single pathway was significantly enriched. This likely reflects a broad, diffuse regulatory response rather than targeted activation of specific metabolic functions. The pronounced growth response of strain A to partner strains suggests that widespread expression changes may obscure individual pathway-level significance.

The other strains – B, C and D – all had pathways differentially overrepresented in response to partners, with amino acid, carbon metabolism and phosphotransferase systems featuring prominently (Figures S15, S16 & S17). Temporal PCA analysis highlights strain C as the dominant driver of transcriptional variation across partner strains (Fig. 4A). Notably, strain D experiences significant downregulation of amino acid biosynthesis, carbon metabolism, phosphotransferase activity, and fructose/mannose metabolism when co-cultured with strain C (Figure S17). In contrast, pyruvate metabolism and glycolysis are upregulated, suggesting metabolic adaptation to competitive stress. The corresponding inhibition of strain D’s growth further supports a resource-driven interaction dynamic (Fig. 2B and C). However, some similar responses are seen in strain B to C, without the corresponding phenotypic inhibition. Comparing strain B grown axenically with strain C paired-culture, valine, leucine and isoleucine biosynthesis pathways are significantly downregulated, and corresponding degradation pathways upregulated (Figure S15). Figure 4B summarises the results of a differential expression analysis of the transcriptomics data for day 4. Each volcano plot shows the differentially expressed genes identified when comparing the gene expression profile of the focus strain grown next to each partner, when compared with the axenic culture. Not surprisingly, the number of differentially expressed genes correlates with the phenotypic changes shown in Fig. 2A. Whilst it is apparent that the expression of core metabolism genes in these strains adjusts to neighbouring bacteria, suggestive of competition for local nutrients as expected, there is no apparent correlation with observed inhibitory phenotypes. Given the presence of tens of BGCs for putative secondary metabolites in each strain, it is reasonably likely antibiotic production drives the observed inhibitions.

### The BGC for the siderophore desferrioxamine is well conserved in the four strains, yet production is highly varied

Manual inspection of the annotated LC-MS dataset revealed an enrichment of desferrioxamine (DFO)-related ions in strain C, with weaker production in strains B and D, and none in strain A. This pattern mirrored preliminary observations that both iron supplementation and commercially sourced desferrioxamine B (DFO-B) could stimulate growth in strain A, prompting us to examine whether DFOs contribute to the interaction phenotypes observed.

**Fig. 5:**
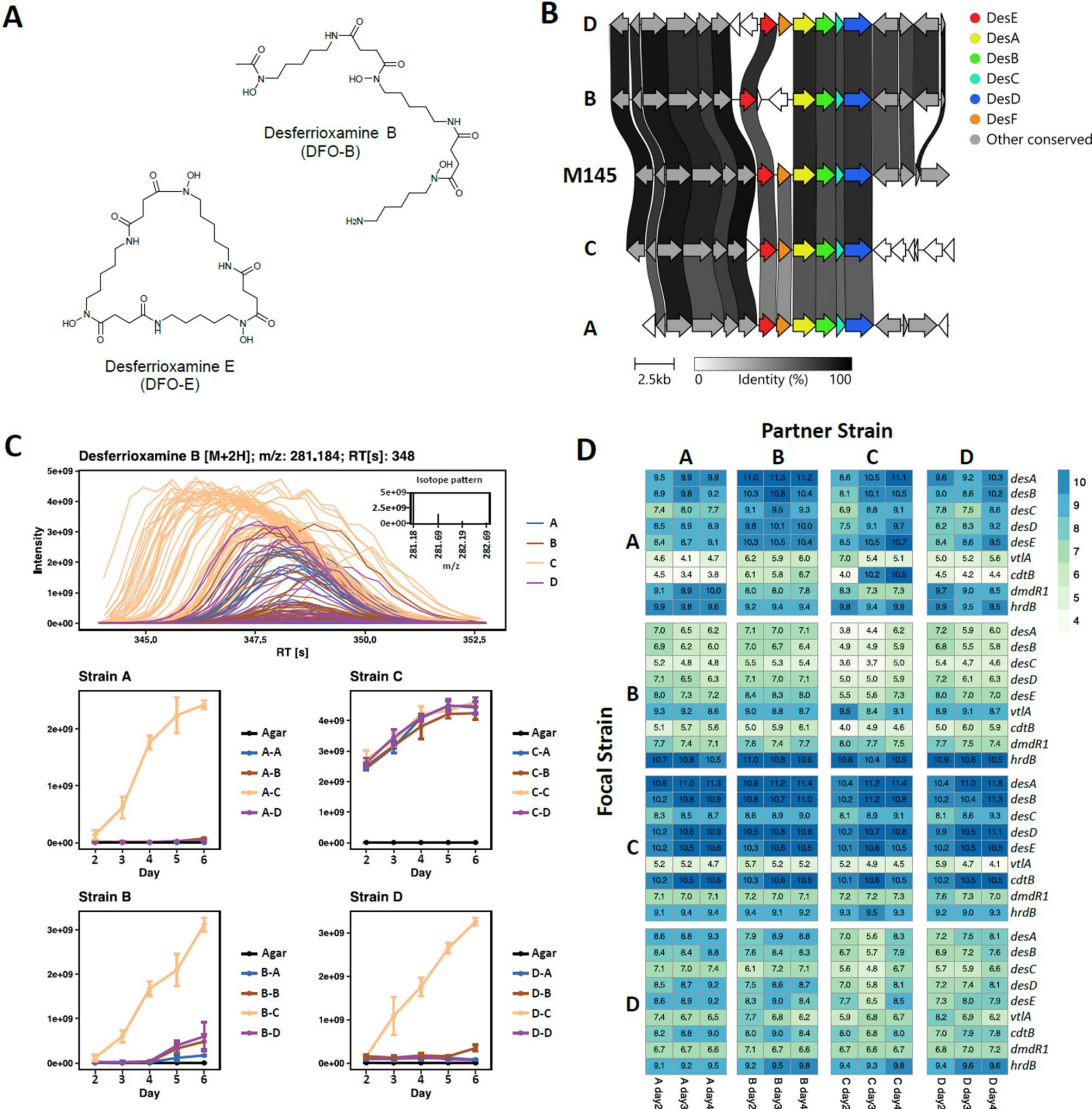
(**A**) Chemical structures of DFO-B and DFO-E. (**B**) Genomic alignment of DFO BGCs, including the model *S. coelicolor* M145. Bars indicate nucleotide identity. The established desferrioxamine BGC is highlighted by the coloured genes. Generated using clinker^46^. (**C**) LC-MS detection of DFO-B. Top panel overlays extracted ion chromatograms showing detected peak shape across the retention time window, and the isotopic spectra. Bottom panels show abundance of ion m/z 281.184 corresponding to DFO-B [M+ 2 H] in each strain combination, including agar controls. (**D**) Heatmap for a curated set of DFO-related genes expression levels and presented in the same grid format as Fig. 3. Expression values shown were obtained using the Median of Ratios method from the DESeq2 package.

Desferrioxamines are a group of related hydroxamate siderophores of clinical importance first discovered in *Streptomyces pilosus*, where they were described as growth factors (“Wuchsstoffe”)^38^. The DFO biosynthetic pathway has been elucidated in *Streptomyces coelicolor* M145, which primarily produces desferrioxamine E (DFO-E, Fig. 5A), as well as DFO-B and related metabolites^39^,^40^. The BGC is well conserved across *Streptomyces*, including the four strains of interest here, where biosynthesis genes *desA*, *desB*, *desC*, *desD* show 81–94% amino acid similarity to their *S. coelicolor* homologs, and are identified by antiSMASH^41^ (Fig. 5B). *desE* encodes a siderophore binding protein involved in ferrioxamine uptake^42^, and is conserved in our strains. However, *desF*, which encodes a putative ferrioxamine reductase, is absent in the BGC of strain B, which instead has ORFs encoding an IS106/110/902 family transposase and a short hypothetical protein. No orthologs to *desF* are present elsewhere in the genome of strain B suggesting it may have an alternative strategy for the release of iron from DFOs.

Here we focus on the linear desferrioxamine B (DFO-B), as its medical use renders it readily available for biochemical assay use. Desferrioxamine E was also identified in the LC-MS analyses (Supplementary Figure S8) but was only available for purchase in small quantities in the ferrioxamine E form, already bound to iron, and therefore less well integrated with the accompanying biochemical tests.

Metabolomic analysis revealed strain C to be the highest DFO-B producer under the conditions tested, with the highest ion counts at all timepoints. DFO-B was also observed from strain B at a lower level from day 5 onwards (Fig. 5C). The metabolomic extraction method used captured both exo- and endo-metabolites, hence the detection of diffusible compounds, such as DFO-B, produced by the partnering strain should be expected. This explains the DFO-B corresponding ions detected in all strains when in combination with the strongly producing C. Notably, no DFO-B was detected from strain A axenic cultures.

A corresponding high level of transcription of the established DFO genes, *desA-E*, was observed in strain C (Fig. 5D). No significant transcription or any increase was observed for strain B by day 4. LC-MS analysis of strain B indicated DFO-B production from day 5. No defects (such as nonsense or missense mutations affecting conserved residues) were apparent in the DNA sequence of the DFO BGC of strain A when aligning to other *Streptomyces* species.

DmdR1 is an iron-regulating transcription factor demonstrated to bind to the iron box operator upstream of *desA*^43^. The corresponding gene and iron boxes are conserved in all four strains. No apparent patterns were observed in *dmdR1* transcription in the strain combinations, although it is notably also highly expressed in strain C, the best DFO producer (Fig. 5D). A second iron-regulating transcription factor, DmdR2 has been described in *S. coelicolor*^43^; however, no orthologs to the corresponding gene are present in the genomes of strains A, B, C and D. DmdR1 is the orthologue of the iron-dependent regulator IdeR from *Streptomyces avermitilis*^27^, and BLAST searches using both proteins return the same single locus in each of our genomes.

*vtlA* encodes a putative VIT1-like exporter that has been implicated as being involved in iron homeostasis in *S. coelicolor*. The encoding gene, *vtlA*, has a DmdR1 operator box upstream, and is part of the CatR regulon^44^. Little expression of *vtlA* was observed in strains A, C and D at any time or partner combination. However, *vtlA* is expressed in strain B, with the DFO BGC largely unexpressed (Fig. 5D). This may be the first indication of an alternative strategy or iron homeostasis state of strain B. The *cdtABC* operon was first implicated in siderophore uptake in pioneering *S. coelicolor* work, where its disruption prevented uptake of salmycin, an antibiotic DFO analogue, yet not DFO-B itself^45^. However, DFO-B was later observed to bind CdtB and DesE in vitro^42^. It is plausible DFO-B uptake is mediated by both in vivo, and this redundancy may explain why *cdtABC* disruption does not prevent DFO-B uptake, however, this remains to be directly confirmed. In strain C, *cdtB* is strongly expressed, which together with strong expression of *desA-E* and DFO production, seems logical. In strain A, when next to the strong DFO producer C, *cdtB* expression is increased 300–350 fold compared with axenic, albeit where the expression is very low. CdtB strongly binds DFO-B and is thought to facilitate its uptake^42^. A less extreme activation of *cdtB* expression was observed with strain A partnered with B (Fig. 5D), which corresponds with its lesser production of DFOs (Fig. 5C). However, *cdtB* expression remains at very low levels. This makes sense in the context of there being no detectable DFO production by B at the earlier timepoints sampled for RNA-seq, and this also supports confounding factors influencing the B-A interaction other than just DFOs.

Regardless of the intriguing implications of the RNA-seq analyses on how the DFO cluster and related gene expression result in DFO-B production, the metabolomics analysis indicates that strain C is a high DFO producer and strain A, a poor one. Therefore, next, biochemical routes were explored to understand if DFOs have a role in this interaction.

### Desferrioxamine B is the major cue underpinning the strain C to A interaction

**Fig. 6:**
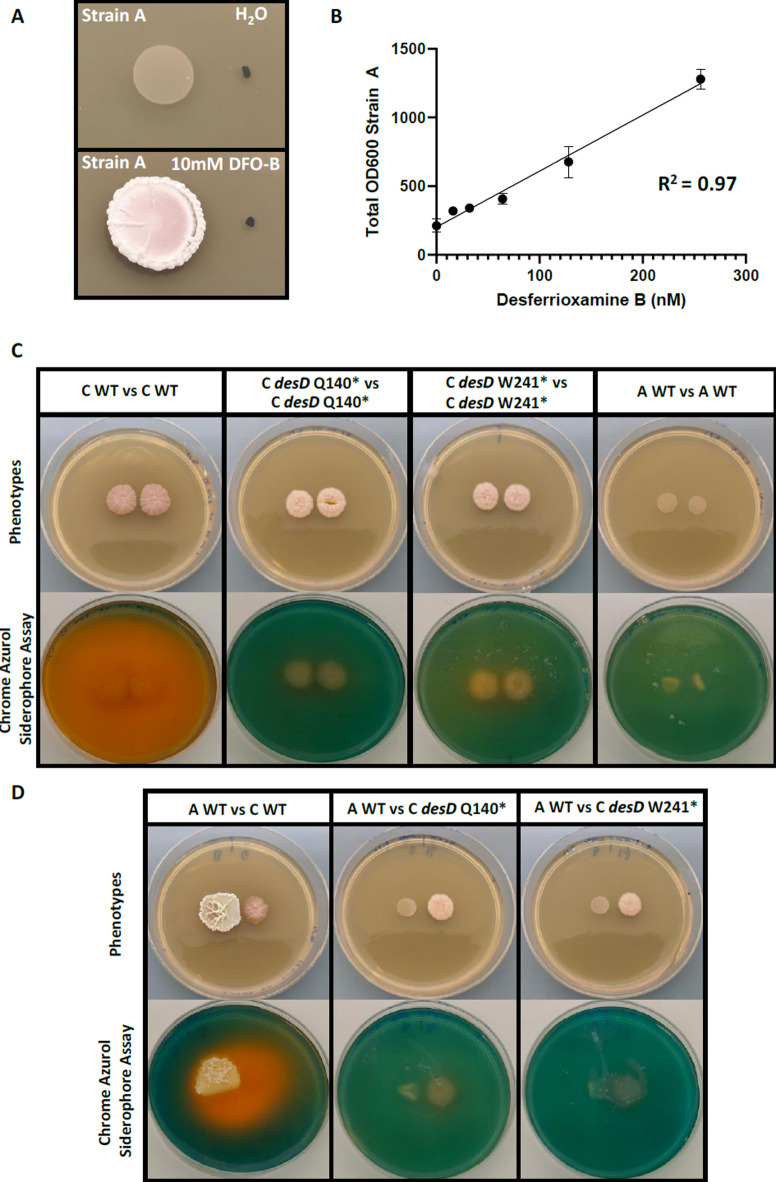
(**A**) Strain A inoculated 1 cm apart from 1 µl spots of water or DFO-B. (**B**) OD600 of strain A measured by well-scan mode in a plate reader of a series of DFO-B dilutions from 256 to 16 nM. Error bars represent standard deviation, *n* = 4. (**C**) Axenic cultures of strain C WT, *desD* nonsense mutations and strain A. The lower panel is the same plates, 16 h post chrome azurol assay overlay. Siderophore production is indicated by the colorimetric change from green to orange. (**D**) Paired cultures of strain A WT, strain C WT and *desD* nonsense mutations. The lower panel are the same plates, 16 h post chrome azurol assay overlay.

Strain A displayed a significant change in phenotype when inoculated next to commercial DFO-B (Fig. 6A). This is reminiscent of its interaction with the highly DFO-B producing strain C, with substantial increases in aerial growth, differentiation and size. Similar morphologies in strain A could be elicited with FeCl_3_ (Supplementary Figure S9).

Strain A was inoculated onto ISP2 agar in a 24-well plate with a serial 2-fold dilution of DFO-B from 256 nM to 16 nM, and a 0 nM control. Growth in the microwell plate allowed in situ measurements of colony size and thickness, with 900 (30 × 30) datapoints per reading per well. The response of strain A to low DFO-B concentrations appeared linear (Fig. 6B). Consistently across all tests performed, strain A demonstrated a strong response to DFO-B, ferrioxamine E and iron chloride (Supplementary Figures S9, S10). These observations, in combination with the consistently high production of DFO-B observed in strain C and its absence in strain A, suggest that DFO-B is likely key to the phenotypic interactions observed between strains C and A.

To test the impact of strain C’s DFO production on the interactions, we aimed to generate a strain C mutant deficient in DFO production. However, strain C failed to accept spCas9 CRISPR plasmids pCMU-4 and pCM-4.4 (not shown), which can be a challenge in non-model environmental isolates such as this^47^. Instead, CRISPR base editing was pursued, where a mutant Cas9 variant nicks a targetable sequence, with a fused cytidine deaminase (in the version used in this work) generating cytidine to thymine mutations across a window. This allowed for the generation of nonsense mutations in the gene of interest.

The core DFO biosynthesis gene *desD* was targeted in strain C; deletion of the orthologous gene in *S. coelicolor* has previously been demonstrated to abolish DFO production^39^. Potential protospacers were screened using CRISPyweb^48^, with manual appraisal of suitability of the editing window based on previous findings^49^, with a preference for specificity and occurring early in the gene to ensure disruption of function. Two protospacers were designed to generate Q140* and W241* in *desD*, cloned to pCRISPR-cBEST and mobilised to strain C (Supplementary Figure S11). Edits were appraised by PCR and sequencing, all four of the exconjugants screened were successful mutants. Both mutants were generated for redundancy; both should disrupt DesD functionality as they result in significant truncations of the 589 amino acid protein.

A similar phenotype to WT was observed for the *desD* mutant strains, with no apparent deficiency in growth on agar (Supplementary Figure S12). This suggests that despite strain C’s high production of DFOs, it does not require DFOs for growth in this setting. Indeed, it seems unlikely that iron would be limiting in the rich ISP2 agar used. Chrome azurol solution (CAS) has been developed as a colorimetric biochemical assay for detection of siderophores, including its proven use for DFO detection. This was replicated in this study first in liquid assays using commercially purchased DFO-B. Colorimetric indication of DFO-B was somewhat apparent 1 h post-application, but significantly better differentiated at 16 h post mixing, where DFO-B was detected to 20 µM (*n* = 2, Supplementary Figure S13).

The application of the CAS siderophore assay as an overlay on agar plates successfully detected strain C’s inherent high siderophore production (Fig. 6C), with the orange zone nearly covering the plate. Both of the *desD* nonsense mutation versions abolished siderophore production as detectable by CAS assay, suggestive that the mutations were successful and that DFOs represent the major component of siderophore activity for strain C in this setting. No siderophore activity was observed for axenic strain A. These biochemical observations align with the DFO production as detected by LC-MS. Finally, the strong induction of growth in strain A by strain C was again observed, and abolished with the inactivation of *desD* in strain C (Fig. 6D). This demonstrates that DFO production in C is the driver of the phenotypic response in strain A, mediating this intriguing interaction between these sympatric environmental strains.

## Discussion

### Desferrioxamine modulates soil microorganism interactions

The remarkable phenotypic change in strain A when grown in combination with strain C, mirrored by large-scale metabolomic and transcriptomic shifts, is consistent with being largely driven by the DFOs produced by strain C. A similar morphological change in strain A was observed with the provision of exogenous DFO-B or FeCl_3_, and deletion of strain C’s DFO biosynthesis completely disrupted the phenotypic response of strain A. This demonstrates that DFOs produced by strain C modulate the fundamental growth of strain A, most likely in a direct fashion. Although it should be noted that indirect effects, where the lack of DFO production in strain C elicits changes in its own metabolome that subsequently also affect strain A, cannot be ruled out.

DFOs have been implicated in eliciting such responses in *Streptomyces* previously, for example exogenous DFO-E but not DFO-B was able to stimulate aerial growth in *S. tanashiensis*^26^. It is unclear why this would occur as both are thought to share biosynthesis, uptake and turnover mechanisms. Here, strain A was extremely responsive to DFO-B, and also to ferrioxamine E (DFO-E was not available) and FeCl_3_. Ferrioxamine E is already bound to iron, which itself elicits a response, hence the preference for DFO-B in the biochemical tests reported here.

It should be noted that high production of both DFO-B and DFO-E was detected from strain C, and that biosynthesis of both will be disrupted by the nonsense mutations of *desD*, as they share biosynthesis enzymes^39^. Thus DFO-E and other closely related hydroxamates produced by the BGC could also contribute to the stimulated growth in strain A in the co-culture. Notably, strain B was the second-best DFO-B producer and elicited the next highest growth response in strain A. DFO production should logically contribute to this interaction also, although the equivalent mutagenesis evidence is not available. However, the metabolomic and transcriptomic changes appear different when grown with strain B compared with strain C, and this may suggest further confounding cues or factors. *Streptomyces* have also been observed to respond with secondary metabolite production and increased antimicrobial activity to another siderophore, catechol^50^. Siderophores have also been implicated in *Streptomyces* responses to fungi^51^^,52^. Chemically diverse siderophores are found in soil^53^^,54^, and it is clear each may likely impact such interactions between microbiome members, which hints at the complexity of ecological and evolutionary interplay within the community. This study goes beyond previous work by showing that DFO-mediated interactions induce large-scale transcriptomic shifts affecting multiple pathways, rather than just altering colony morphology. The integration of KEGG pathway enrichment analysis allows us to pinpoint functional changes, providing a deeper mechanistic insight into microbial cross-talk.

### Alternative evolutionary strategies for iron chelation competition

DFOs provide an interesting opportunity to compare iron acquisition strategies between the environmental strains, given each shares a conserved DFO BGC (Fig. 5A). Strain C constitutively produces high levels of DFOs, yet it does not require any DFO production to grow in a similar fashion on this rich medium (Fig. 6C). No other siderophore activity was detected by CAS overlay assay. While in our study and others^55^^,56^, excess siderophores can be used by competitors, excess siderophore production can be a useful strategy for competing. A ‘locking away’ evolutionary strategy has been described in which excess siderophore is produced to chelate iron and render it inaccessible to competitors^20^. Competition between *Streptomyces* and pathogenic fungi has been observed in both directions, where locking away of bioavailable iron via siderophore production inhibits the growth of a competitor^25,^^,57^^,58^,. This explains why the excessive siderophore production of strain C may have evolved.

Strain B was observed to produce significantly less DFO-B, and at a later time point. This could be a straightforward response to decreasing iron availability as the culture establishes, or it might be tied to global developmental regulation. DFO and siderophore production itself has been observed responsive to external cues and competitors^59^^,60^; however, no changes in DFO production were observed in the metabolomics with respect to partner strains. An inherent limitation of exometabolomics, coupled with the shared ability of all strains to synthesise DFOs, means that diffusion of the high levels of DFOs produced by strain C could mask changes in any partner strain of C’s. Strain A did show a significant upregulation of DFO biosynthesis genes when cultured with strains C and B, with no corresponding detection on LC-MS subject to these caveats.

### Strain A exhibits siderophore piracy but does not conform to a classical cheating strategy

At a superficial level, strain A fits the evolutionary strategy classification as a cheat, defined as an individual that exploits communal public goods by benefiting from their production without bearing any of the associated metabolic costs^20^. It also employs siderophore piracy, defined as the uptake of iron-chelating siderophores secreted by other organisms via dedicated receptors, thereby avoiding the metabolic costs of siderophore biosynthesis^25^. It appears to avoid the expense of DFO production and instead relies on the DFO produced by others. The strains were isolated from the same soil core, and therefore strain A may have evolved with the DFO-B-producing strains B and C, as well as any other DFO producers in the microbiome, making a pirating strategy viable. However, RNA-seq revealed that the core DFO biosynthesis genes *desABCD* are transcribed at similar levels to strain D and higher levels than strain B. One possible explanation for the lack of DFO production in strain A despite BGC expression is the lack of available chemical precursors. DFO biosynthesis utilises L-lysine, acetyl-CoA (DFO-B only), and succinyl-CoA (both DFO-B and DFO-E)^40^. However, it does not seem plausible that such key primary metabolites are completely unavailable for DFO biosynthesis.

The CAS assays, LC-MS analysis and phenotypic data were all consistent in supporting the observation that strain A produces no DFOs or other siderophores, despite requiring them to stimulate its own growth. It remains perplexing why strain A would be such a strict cheat as to prevent its own proliferation, although perhaps the observed behaviour is an artefact of the defined laboratory experiment environment, as opposed to the much more diverse and competitive soil microbiome. Indeed, it is also strange that strain A would require exogenous iron or siderophores to stimulate growth on this rich medium at all, where one would not expect iron to be limiting. This is supported by the observation that strain C grows similarly to its siderophore-deficient *desD* mutants when each is grown axenically.

Upon culturing with strain C, strain A appears to repay the stimulation of its growth with inhibition of strain C, with a directional inhibition observed on the interaction plates (Fig. 2C and Supplementary Figure S14). One potential explanation would be that the piracy of siderophores inhibits strain C’s growth through iron starvation; such siderophore piracy-mediated growth inhibition has previously been observed from *Amycolatopsis* to *Streptomyces*^25^. That strain C with the DFO mutations which grows well, does not support this, although it should be noted that successful growth in the absence of a siderophore is not directly equivalent to growth when the siderophore is produced and pirated by a competitor. In the latter scenario, the produced siderophore is chelating and effectively removing iron from the shared environment as it is pirated. There has been a report of MALDI-TOF imaging of DFOs^24^, and such an approach could be telling as to the interface of strains C and A in a 2-D plane. Another plausible explanation is that DFO produced by strain C stimulates the observed large-scale metabolic and morphological changes in strain A, part of which could include the production of antimicrobials. This is supported by the activation of secondary metabolite BGCs as detected by RNA-seq (Supplementary Figure S1). Corresponding metabolites have not been identified (or inferred to be absent); this would represent a further challenge where BGCs are novel. Further work, such as testing extracts of strain A grown under different iron conditions for inhibitory activity against strain C, could help clarify whether secreted metabolites contribute to this effect.

Overall, these findings demonstrate that microbial interactions mediated by DFOs extend beyond simple iron acquisition, shaping both metabolic and phenotypic responses. By combining transcriptomic and metabolomic analyses with quantitative measurements, we establish a robust approach for investigating siderophore-mediated interactions in microbial communities. Our results highlight the role of DFOs as inter-species cues, triggering substantial phenotypic and molecular changes in responsive strains. Through a multimodal analysis incorporating transcriptomics, metabolomics, and automated colony measurements, we present the response of bacteria to their neighbours at the molecular level. This methodological framework strengthens the foundation for future research in microbial ecology, synthetic biology, and microbial consortia dynamics.

## Materials and methods

### Cultivation of strains and media

All strains, plasmids and primers used in this study are listed in Supplementary Tables S2, S3 and S4 respectively. For general cultivation, strains were incubated on SFM or ISP2 agar at 30 °C, conjugations were performed on SFM agar. All interaction, molecular profiling and desferrioxamine experiments were performed on ISP2 agar. SFM was prepared per litre of tap water: 20 g soya flour (Holland & Barrett), 20 g mannitol (Fisher), 20 g agar (Fisher). ISP2 was prepared per litre distilled water: 4 g yeast extract (Oxoid), 10 g malt extract (Difco), 4 g D-glucose (Fisher), optionally 20 g agar, with the pH adjusted to 7.2 by 2 M NaOH.

To culture the interacting bacteria for molecular profiling analysis, spores of each strain had first been standardised and aliquoted at 1 × 10^9^ CFU ml^− 1^ in 20% glycerol. Before use, these were diluted 10-fold to 1 × 10^8^ CFU ml^− 1^ in ISP2 broth, and 4 µl inoculated 1 cm apart in pairs on ISP2 agar. Each pairing was prepared in quadruplicate for each of the 5 timepoints (*n* = 20 total for each pairing), to allow for the metabolite and RNA sampling in triplicate daily (with one spare). One set of quadruplicate plates was chosen at random to be photographed daily, such that the same four plates were imaged to capture phenotypes, considering our sampling on earlier timepoints is destructive. Images were captured daily on days 2 through 6 of the quadruplicate set.

### **Taxonomic** assignment of isolates

The taxonomic classification of bacterial isolates was performed using multilocus sequence typing with autoMLST^30^ against NCBI Refseq. Average nucleotide identity to the closest reference genomes was determined using FastANI^61^.

### Colony area measurements and analysis

Bacterial colony areas were quantified from digital images of Petri dishes using custom Python scripts leveraging the OpenCV (https://github.com/itseez/opencv) and imutils (https://github.com/PyImageSearch/imutils) libraries. For each image, the analysis proceeded as follows: The image was first converted to grayscale. Colony segmentation was achieved by applying a binary threshold to isolate the colony pixels from the background. Contours representing distinct objects within the thresholded image were detected using the cv2.findContours function, further processed by imutils.grab_contours. The area (in pixels) of each detected contour was calculated using cv2.contourArea.

Pixel areas were converted to square millimetres (mm²) using a calculated conversion factor derived from the known size of the plate. Statistical comparisons of colony areas between different partner conditions for each primary strain were performed using pairwise two-sample t-tests. P-values were adjusted using the Benjamini-Hochberg correction method. A Tukey’s Honest Significant Difference test was also performed and found not to change the biological interpretation of the significant interactions (Figure S18).

### Construction of CRISPR plasmids

To prepare the base-editing plasmid for protospacer cloning, pCRISPR-cBEST^62^ was linearised with NcoI-HF (NEB), and dephosphorylated with Quick CIP (NEB). Protospacers were identified and checked for predicted genome-wide specificity with CRISPyweb^48^, with manual selection of good editing windows based on previously described efficiencies^49^. Two redundant base editing designs were generated: the first protospacer was designed to edit W241* in *desD* on the Strain C genome, the second for Q140* also in *desD*. Base editing plasmids were constructed by NEBuilder Hifi Assembly (NEB) between single-stranded oligonucleotides and *Nco*I linearised pCRISPR-cBEST (Supplementary Figure S11).

### Inactivation of *desD* by CRISPR base editing

Plasmids were conjugated to *Streptomyces* by intergeneric conjugation following established protocols^62^ from *E. coli* ET12567/pUZ8002, selecting for apramycin (50 µg ml^–1^). Exconjugants were restreaked to SFM nalidixic acid (15 µg ml^–1^) and apramycin (50 µg ml^–1^), before screening by colony PCR with primers oJC136 and oJC137. PCR products were Sanger sequenced (Eurofins genomics) in both directions to confirm mutant *desD* sequences.

### Chrome azurol assay for siderophore activity

The Chrome azurol S assay (CAS) is an established method of siderophore detection^63^. To prepare chrome azurol assay solution for siderophore assay, 50 ml of 2 mM chrome azurol S (Fisher Scientific) in ddH_2_O was mixed with 9 ml of 1 mM FeCl_3_ in 10 mM HCl, and subsequently mixed with 40 ml of 5 mM hexadecyltrimethylammonium bromide (HDTMA, Thermo Scientific) in ddH_2_O^64^. For liquid assays, a ratio of 1:10 of CAS solution to final volume sample solution was used. For example, 10 µl CAS solution was added to 90 µl of 2.22 mM DFO-B (Thermo Scientific) in water to test detection of 2 mM DFO-B. Test solutions were incubated for 16 h at 30 °C before imaging.

For plate-based assays, the previously described CAS agar assay utilised an alternative medium reported unsuitable for Gram positive bacteria. To allow assay on the same ISP2 agar as our experiments, a CAS overlay was instead applied, in a similar fashion to O-CAS^65^. To prepare the overlay, 100 mM MOPS (3-(N-morpholino)propanesulfonic acid, Merck) was adjusted to pH 7.2, and autoclaved with 1.1% (w/v) agar. As before, CAS solution was added at a 1:10 ratio to the final volume of CAS agar overlay. The CAS-agar solution (15 ml) was overlaid onto the ISP2 agar plates with established *Streptomyces* growth and incubated at 30 °C for 16 h before imaging.

### Plate reader assay for cell growth in a microwell on media supplemented with DFO-B

The experiment was performed in a 24-well microplate (Corning) in quadruplicate. ISP2 agar with 256, 128, 64, 32, 16 and 0 nM DFO-B was prepared from a 20 mM DFO-B stock in H_2_O, with 500 µl aliquoted per well. Plates were inoculated with 1 µL of 1 × 10^8^ CFU ml^− 1^ strain A spores, prepared by ten-fold dilution of 1 × 10^9^ CFU ml^− 1^ stocks in ISP2 broth. The plate was incubated at 30 °C, and OD600 measured in a Clariostar plate reader (BMG) daily in well-scan mode, with the matrix resolution set to the maximum of 900 readings per well (30 × 30).

### RNA isolation

*Streptomyces* mycelia were scraped from the agar surface with a spatula, and frozen in liquid nitrogen before storage at −80 °C. Frozen cells were transferred to Lysing Matrix E 2 ml tubes (MP Biomedicals LLC) before lysis with a FastPrep bead homogeniser (MP Biomedicals LLC) set to 6.0 m/s for 20 s. Trizol (1 ml, ThermoFisher) was added to each tube, mixed by hand inversion and incubated for 5 min to allow dissociation of the nucleoprotein complex. Samples were centrifuged at 16,000 x g for 10 min, and 700 µl of supernatant was collected. To each, 140 µl of chloroform was added with incubation at room temperature for 3 min with occasional mixing by hand. After centrifugation for 15 min at 12,000 x g at 4 °C, samples separate into a lower red phenol-chloroform, interphase and colourless upper aqueous phase. The aqueous phase (2 × 140 µl) containing the RNA was transferred to a fresh tube, before removal of 200 µl with the tube tilted to avoid any contamination of the other phases. An equal volume of 200 µl 100% of ethanol was added to each.

RNA isolation from the resulting samples was performed using the Direct-zol RNA Miniprep kit (Zymo Research) with the manufacturer’s protocol, omitting the on-column DNase treatment (that had proven ineffective in this experiment as indicated by qPCR). Samples were eluted in 50 µl autoclaved milli-Q water and concentration monitored by Qubit HS and BR RNA assay kits (Thermofisher). To remove residual gDNA in 50 µl samples, the manufacturer’s protocol was followed for TURBO DNase kit (Ambion), with 1 µl DNase per 50 µl input sample, including the DNase removal steps by kit reagent and centrifugation. RNA integrity was measured by recording RINs with a Bioanalyzer 2100 (Agilent).

### RNA-seq

Library preparation and sequencing was performed by Source Bioscience. The libraries were prepared with the NEBNext rRNA Depletion Kit for Bacteria (NEB) according to the manufacturer’s protocol. During this process, the libraries were indexed with NEBNext Multiplex Oligos for Illumina (NEB). Illumina sequencing was performed at depth 30 million read pairs per sample as 150 bp paired end reads.

RNA samples were multiplexed before library generation and subsequent sequencing. To confirm our assertion that all four strains could be multiplexed without significant data loss, preliminary RNA-seq was performed with samples from strains B and C run individually, compared with the same samples mixed with each other and all four strains. No significant changes were observed in gene expression due to multiplexing, and therefore this strategy was applied to all further RNA-seq. Samples were multiplexed as detailed in Supplementary Table S9.

### RNA-seq analysis

To reduce the total number of RNA samples a multiplexing approach was used. This required developing an ad hoc data processing pipeline for the resulting data. Fastq files containing the raw reads were processed with the BBDuk and BBMap bioinformatics tools (B. Bushnell, sourceforge.net/projects/bbmap/). On a first run, BBDuk was used to trim the adapter sequences from the reads. On a second run, BBDuk was used to perform an rRNA decontamination step. For this step the SILVA database^66^ was modified by including the rRNA sequence from the strains considered in this study. The BBMap tool was then used to map the remaining reads to the four concatenated genomes considered in this study. Reads with ambiguous matches across the four genomes were discarded. The alignment data obtained together with the concatenated genomes were then processed using the Bioconductor packages Rsamtools, GenomicFeatures and GenomicAlignments^67^ to obtain the raw counts data table. The transcript per million (TPM) values were obtained from an in-house developed R script following the procedure described previously^68^. Differential expression analysis was performed with the Bioconductor R package DESeq2^34^.

### Pathway enrichment analysis

Differential gene expression analysis between conditions was performed using the R package DESeq2^34^ with a likelihood ratio test (LRT) considering the experimental design factors ‘partner’ and ‘day’, and their interaction. Genes were considered significantly differentially expressed if the Benjamini-Hochberg adjusted p-value was ≤ 0.05 and the absolute log_2_ fold change was > 1.

Functional enrichment analysis of the resulting upregulated and downregulated gene sets was conducted separately using the R package clusterProfiler^69^. Gene annotations, specifically KEGG^35,36^^37^ Orthology (KO) identifiers, were obtained using eggNOG-mapper^70^ and processed into a format suitable for clusterProfiler. KEGG pathway enrichment was performed using the enrichKEGG function with KO identifiers. The analysis employed a hypergeometric test, considering pathways with a minimum gene set size of 10. Significance was determined based on a p-value cutoff of 0.05, adjusted for multiple comparisons using the Benjamini-Hochberg method.

### Metabolite extraction

Rectangular agar plugs (1 × 0.5 cm) were excised, including the *Streptomyces* mycelia and the surrounding agar, and stored at − 80 °C. Samples were thawed on ice and 1 ml of 100% methanol precooled to − 48 °C added to each. Three freeze–thaw cycles were performed between 1 min in liquid nitrogen, thawing on ice and mixing by vortex. Samples were centrifuged at 8000 x g for 10 min at 4 °C, and the 1 ml supernatant collected and dried by SpeedVac at room temperature (Thermo Fisher). Prior to injection, samples were reconstituted in 200 µl of 20% methanol, sonicated for 5 min, and passed through a 0.2 μm filter (F2517-5, Thermo Fisher).

### LC-MS data acquisition

Samples were analysed on a Q Exactive Plus coupled to an Ultimate 3000 UHPLC (Thermo Fisher) equipped with a Hypersil GOLD C18 reversed phase HPLC column (3 μm, 2.1 mm, 100 mm; 25003–102130- Thermo Fisher). The mobile phase consisted of solvent (A) water + 0.1% formic acid and solvent (B) methanol + 0.1% formic acid. The flow gradient was programmed to equilibrate at 95% A for 2 min, followed by a linear gradient to 95% B over 8 min, held at 95% B for 2 min, then followed by a return to 95% A in 0.25 min and held at 95% A for further 2 min. The column was maintained at 40 °C and samples chilled in the autosampler at 4 °C. The flow rate was set to 0.4 ml min^–1^. The sample injection volume was 5 µL. Blank injections were analysed at the start and end of the analytical batch to assess the carryover. In addition, pooled quality control (QC) samples were analysed at every 6th injection to assess for analytical drift over time. The sample sequence was randomised. Data were acquired in full MS mode in the scan range of 90–1350 m/z, with a resolution of 70,000, an AGC target 3e6 and a maximum integration time of 200 ms. The samples were analysed in positive and negative mode in separate acquisitions.

### LC-MS data analysis

Raw data files from the Q Exactive were converted into the mzML format by the ProteoWizard MS converter. Data analysis was performed with the use of mzMatch, a modular, open-source, and platform-independent data processing pipeline for metabolomics LC-MS data written in the Java language implemented in R^71^. Noise removal, signal filtering, and peak matching steps were performed. The detected features were grouped according to their likelihood to be associated with one single molecule, and only the most intense peak is considered for the subsequent statistical analysis. Putative annotation for the detected features was performed with the Integrated Probabilistic Annotation (IPA)^32^^,33^, using the built-in database updated with the information gathered for the analysis of different standards, as described in the tool’s documentation. It should be noted that this method has been developed in our group and therefore the built-in database was specifically constructed around the same experimental settings and instrumentation used in this work. Upstream of the statistical analysis, the following pre-processing steps were applied to the data: signal drift and batch-effect correction, probabilistic quotient normalization, missing values imputation through the k-nearest neighbour method and variance stabilization through the generalised logarithm (glog) transformation. The effect of the pre-processing pipeline is shown in Supplementary Figure S7. All these steps were performed using the pmp Bioconductor R package^72^.

For the selection of peaks produced by different strains in response to their partner the following method was used. When looking for peaks produced by A in response to C, a t-test was used to compare the sample of A growing next to C (A-C) with the sample of A growing next to itself (A-A) and with the sampling of C growing next to A (C-A). Features showing a statistically significant difference (*p*_*adj*_ < = 0.05 and log_2_FC > = 1.5) in at least one time point were selected. P-values were corrected for multiple testing using the Benjamini-Hochberg method^73^.

## Supplementary Information

Below is the link to the electronic supplementary material.

Supplementary Material 1

## Data Availability

Plasmid sequences and DNA have been deposited with Addgene, https://www.addgene.org/219650/ for pTE1643, https://www.addgene.org/219651/ for pTE1644. Datasets have been deposited with the Environmental Information Data Centre (NERC): phenotypic photographs (DOI: 10.5285/3f48eb4c-bc0d-49e3-af71-dcff6d88c534), analysed metabolic profiles (DOI: 10.5285/4921f306-5efa-4bf7-a15e-a157c2665889), raw metabolic data (DOI:10.5285/475d6c02-45bb-4ac4-a31a-4a23c7c9cb2e). Genomes are available on the JGI Genome Portal: strain A (genome ID 2863083887), strain B (genome ID 2781126040), strain C (genome ID 2758568662), and strain D (genome ID 2675903008). The RNAseq datasets are available at NCBI Gene Expression Omnibus (Geo) with accession GSE318666.
