# Supplementary material for: Multi-omics analysis of interspecies interactions in a soil Streptomyces community provides functional insights into siderophore ecology
Source: Sci Rep. 2026 Apr 6;16:11742. doi: 10.1038/s41598-026-45368-6 (PMC13061897; doi:10.1038/s41598-026-45368-6)
Supplement: Supplementary file 1 — Supplementary Material 1 [file 41598_2026_45368_MOESM1_ESM.docx]

**Multi-omics analysis of interspecies interactions in a soil *Streptomyces* community provides functional insights into siderophore ecology**

Connolly, J. A. ^# A^, Del Carratore, F.^# B A^, Schmidt, K.^A C^, Bisesi, A.^D^, Martinson J. N. V. ^D E^, Chua, J. ^A^, Kuhs, M.^D^, Boneza, M.^F^, Heinsch, S.^F^, Kinkel, L.^G^, Smanski, M.^F H^, Harcombe, W.R.^D H^, Breitling, R. ^A^, Takano, E^* A^.

^A^ Manchester Institute of Biotechnology, Department of Chemistry, School of Natural Sciences, Faculty of Science and Engineering, University of Manchester, Manchester, M1 7DN, United Kingdom
^B^ Department of Biochemistry, Cell and Systems Biology, Institute of Integrative, Systems and Molecular Biology, University of Liverpool, Liverpool, L69 3BX, United Kingdom
^C^ Division of Immunology, Immunity to Infection and Respiratory Medicine, University of Manchester, Manchester, United Kingdom
^D^ Department of Ecology, Evolution and Behaviour, University of Minnesota, St. Paul, Minnesota, USA
^E^ Innovative Genomics Institute, University of California, Berkeley, California, USA
^F^ Department of Biochemistry, Molecular Biology, and Biophysics, University of Minnesota, St. Paul, Minnesota, USA
^G^ Department of Plant Pathology, University of Minnesota, St. Paul, Minnesota, USA
^H^ Biotechnology Institute, University of Minnesota, St. Paul, Minnesota, USA
^*^ Corresponding author
^#^ Contributed equally

**Supplementary Tables and Figures**

**Contents**

[Table S1 – Genes used to generate phylogeny with AutoMLST ^1^. 3](#_Toc224313333)

[Table S2 – Bacterial strains used in this study. 5](#_Toc224313334)

[Table S3 – Plasmids used in this study, with Addgene repository numbers. 5](#_Toc224313335)

[Table S4 – Oligonucleotides used in this study. 5](#_Toc224313336)

[Table S5 – AntiSMASH summary output of predicted BGC of Strain A 6](#_Toc224313337)

[Table S6 – AntiSMASH summary output of predicted biosynthetic gene clusters of Strain B 8](#_Toc224313338)

[Table S7 – AntiSMASH summary output of predicted biosynthetic gene clusters of Strain C 9](#_Toc224313339)

[Table S8 – AntiSMASH summary output of predicted biosynthetic gene clusters of Strain D 12](#_Toc224313340)

[Table S9 – Transcriptomic sample labelling and multiplexing. Samples were multiplexed into 4 mixes per replicate (n=3) per day (days 2, 3 and 4). Resulting in 36 multiplexed RNA-Seq samples. 14](#_Toc224313341)

[Figure S1 – Significant changes in expression of strain A core BGC genes in response to partner strains (right label), relative to axenic cultures. Top labels correspond to sampling day 2, 3 or 4. Summary information on the corresponding BGCs is presented in Table S5. 15](#_Toc224313342)

[Figure S2 – Significant changes in expression of strain B core BGC genes in response to partner strains (right label), relative to axenic cultures. Top labels correspond to sampling day 2, 3 or 4. Summary information on the corresponding BGCs is presented in Table S6. 16](#_Toc224313343)

[Figure S3 – Significant changes in expression of strain C core BGC genes in response to partner strains (right label), relative to axenic cultures. Top labels correspond to sampling day 2, 3 or 4. Summary information on the corresponding BGCs is presented in Table S7. 17](#_Toc224313344)

[Figure S4 – Significant changes in expression of strain D core BGC genes in response to partner strains (right label), relative to axenic cultures. Top labels correspond to sampling day 2, 3 or 4. Summary information on the corresponding BGCs is presented in Table S8. 18](#_Toc224313345)

[Figure S5 – Heatmap of expression of iron-related genes filtered by annotation containing ‘ferr’, ‘iron’, ‘sidero’, ‘heme’, ‘haem’, ‘hemo’ and ‘fur’. Significantly (P<0.05) upregulated genes are indicated red, downregulated indicated blue. 19](#_Toc224313346)

[Figure S6 – Heatmaps of the normalised intensities associated with ions likely produced by the different strains. Ions detected in positive mode are on the left and negative mode on the right. Each column represents one sampling day, and each group of columns represent the partner strain. Putative compound annotations are included here, where ipaPy2 gives a posterior probability > 0.6. 20](#_Toc224313347)

[Figure S8 – LC-MS detection of DFO-E. Top panel overlays extracted ion chromatograms showing detected peak shape across the retention time window. Bottom panels show abundance of ion m/z 601.3558 corresponding to DFO-E [M+H] in each strain combination, including agar controls. 22](#_Toc224313348)

[Figure S9 – Response of Strain A to an iron chloride gradient. Strain A was incubated for 3 days at 30 °C on an ISP2 agar plate prepared with slanted layers of 0 and 250 nM FeCl_3_ to give a gradient approximately as indicated. 23](#_Toc224313349)

[Figure S10 – Strain A spotted 1 cm away from indicated compounds, after 4 days incubation at 30 °C, n=3. 23](#_Toc224313350)

[Figure S11 – Overview of base editing CRISPR plasmid designs. Plasmids were constructed by NEBuilder Hifi Assembly into pCRISPR-cBEST linearised by *Nco*I digest and dephosphorylated with Quick CIP (NEB). Full oligonucleotide sequences are listed in Table S4. 24](#_Toc224313351)

[Figure S12 – A) Daily phenotype photos of Strain C WT axenic pairs. The bottom row includes 50 µM DFO-B throughout the agar, the top row has none. B) Daily phenotype photos of Strain C *desD* W241* DFO-B biosynthesis mutants. The bottom row includes 50 µM DFO-B throughout the agar, the top row has none. 25](#_Toc224313352)

[Figure S13 – Chrome azurol solution control assay of DFO-B standards. 26](#_Toc224313353)

[Figure S14 – Zoomed photos of the interaction of strains A and C. Guidelines are added to help visualise the apparent directional inhibition of Strain C by Strain A, solid lines represent where a symmetrical colony would reach, dashed lines represent approximately where the colony border is. The top right photo is the same but unmarked. 27](#_Toc224313354)

[Figure S15 – Enrichment analysis of **strain B** – KEGG pathways up/downregulated in the RNAseq data. Each panel shows pathways differentially expressed in strain B in the comparison given in the subtitle. For instance, the A vs D upregulated panel shows pathways upregulated in strain B when next to D compared with when next to A. Where comparisons are absent, e.g. A vs B, the analysis showed no pathways enriched. 28](#_Toc224313355)

[Figure S16 – Enrichment analysis of **strain C** – KEGG pathways up/downregulated in the RNAseq data. Each panel shows pathways differentially expressed in strain C in the comparison given in the subtitle. For instance, the A vs D upregulated panel shows pathways upregulated in strain C when next to D compared with when next to A. Where comparisons are absent, e.g. B vs D, the analysis showed no pathways enriched. 30](#_Toc224313356)

[Figure S17 – Enrichment analysis of **strain D** – KEGG pathways up/downregulated in the RNAseq data. Each panel shows pathways differentially expressed in strain D in the comparison given in the subtitle. For instance, the B vs C upregulated panel shows pathways upregulated in strain D when next to C compared with when next to B. 31](#_Toc224313357)

[Figure S18 – Comparison of multiple testing correction methods for pairwise comparisons of computed strain areas (comparative to Figure 2B). Areas were determined algorithmically on day 6, n=4. The manuscript uses multiple t-tests with a Benjamini-Hochberg correction for multiple testing (left panel). Tukey’s Honest Significant Difference test (right panel) does not change the biological interpretation of the significant interactions. Significance levels are indicated as follows: **** = P <0.0001 *** = *P* < 0.001, ** = *P* < 0.01, * = *P* < 0.05 32](#_Toc224313358)

[Supplementary Material References 33](#_Toc224313359)

# Table S1 – Genes used to generate phylogeny with AutoMLST ^1^.

| **TIGRFAM number** | **Gene** | **Biological process** | **Product Name** |
| --- | --- | --- | --- |
| TIGR00133 | gatB | Protein synthesis | aspartyl/glutamyl-tRNA(Asn/Gln) amidotransferase, B subunit |
| TIGR00019 | prfA | Protein synthesis | peptide chain release factor 1 |
| TIGR03953 | rplD_bact | Protein synthesis | 50S ribosomal protein uL4 |
| TIGR01855 | IMP_synth_hisH | Amino acid biosynthesis | imidazole glycerol phosphate synthase, glutamine amidotransferase subunit |
| TIGR01959 | nuoF_fam | Energy metabolism | NADH oxidoreductase (quinone), F subunit |
| TIGR01694 | MTAP | Purines, pyrimidines, nucleosides, and nucleotides | methylthioadenosine phosphorylase |
| TIGR00138 | rsmG_gidB | Protein synthesis | 16S rRNA (guanine(527)-N(7))-methyltransferase RsmG |
| TIGR02673 | FtsE | Cellular processes | cell division ATP-binding protein FtsE |
| TIGR00708 | cobA | Biosynthesis of cofactors, prosthetic groups, and carriers | cob(I)yrinic acid a,c-diamide adenosyltransferase |
| TIGR00150 | T6A_YjeE | Protein synthesis | tRNA threonylcarbamoyl adenosine modification protein YjeE |
| TIGR01529 | argR_whole | Regulatory functions | arginine repressor |
| TIGR00174 | miaA | Protein synthesis | tRNA dimethylallyltransferase |
| TIGR00036 | dapB | Amino acid biosynthesis | 4-hydroxy-tetrahydrodipicolinate reductase |
| TIGR01632 | L11_bact | Protein synthesis | ribosomal protein uL11 |
| TIGR00647 | DNA_bind_WhiA | Cellular processes | DNA-binding protein WhiA |
| TIGR01083 | nth | DNA metabolism | endonuclease III |
| TIGR00577 | fpg | DNA metabolism | DNA-formamidopyrimidine glycosylase |
| TIGR00763 | lon | Protein fate | endopeptidase La |
| TIGR02970 | succ_dehyd_cytB | Energy metabolism | succinate dehydrogenase, cytochrome b556 subunit |
| TIGR03705 | poly_P_kin | Central intermediary metabolism | polyphosphate kinase 1 |
| TIGR00518 | alaDH | Energy metabolism | alanine dehydrogenase |
| TIGR01169 | rplA_bact | Protein synthesis | ribosomal protein uL1 |
| TIGR02127 | pyrF_sub2 | Purines, pyrimidines, nucleosides, and nucleotides | orotidine 5'-phosphate decarboxylase |
| TIGR01798 | cit_synth_I | Energy metabolism | citrate (Si)-synthase |
| TIGR00482 | TIGR00482 | Biosynthesis of cofactors, prosthetic groups, and carriers | nicotinate (nicotinamide) nucleotide adenylyltransferase |
| TIGR03654 | L6_bact | Protein synthesis | ribosomal protein uL6 |
| TIGR00338 | serB | Amino acid biosynthesis | phosphoserine phosphatase SerB |
| TIGR01994 | SUF_scaf_2 | Biosynthesis of cofactors, prosthetic groups, and carriers | SUF system FeS assembly protein, NifU family |
| TIGR00420 | trmU | Protein synthesis | tRNA (5-methylaminomethyl-2-thiouridylate)-methyltransferase |
| TIGR01066 | rplM_bact | Protein synthesis | ribosomal protein uL13 |
| TIGR03800 | PLP_synth_Pdx2 | Biosynthesis of cofactors, prosthetic groups, and carriers | pyridoxal 5'-phosphate synthase, glutaminase subunit Pdx2 |
| TIGR01816 | sdhA_forward | Energy metabolism | succinate dehydrogenase, flavoprotein subunit |
| TIGR01063 | gyrA | DNA metabolism | DNA gyrase, A subunit |
| TIGR01127 | ilvA_1Cterm | Amino acid biosynthesis | threonine ammonia-lyase |
| TIGR00431 | TruB | Protein synthesis | tRNA pseudouridine(55) synthase |
| TIGR01024 | rplS_bact | Protein synthesis | ribosomal protein bL19 |
| TIGR00168 | infC | Protein synthesis | translation initiation factor IF-3 |
| TIGR00042 | TIGR00042 | DNA metabolism | non-canonical purine NTP pyrophosphatase, RdgB/HAM1 family |
| TIGR00337 | PyrG | Purines, pyrimidines, nucleosides, and nucleotides | CTP synthase |
| TIGR01966 | RNasePH | Transcription | ribonuclease PH |
| TIGR00554 | panK_bact | Biosynthesis of cofactors, prosthetic groups, and carriers | pantothenate kinase |
| TIGR00065 | ftsZ | Cellular processes | cell division protein FtsZ |
| TIGR01224 | hutI | Energy metabolism | imidazolonepropionase |
| TIGR00048 | rRNA_mod_RlmN | Protein synthesis | 23S rRNA (adenine(2503)-C(2))-methyltransferase |
| TIGR00436 | era | Protein synthesis | GTP-binding protein Era |
| TIGR02729 | Obg_CgtA | Protein synthesis | Obg family GTPase CgtA |
| TIGR00062 | L27 | Protein synthesis | ribosomal protein bL27 |
| TIGR01394 | TypA_BipA | Regulatory functions | GTP-binding protein TypA/BipA |
| TIGR00020 | prfB | Protein synthesis | peptide chain release factor 2 |
| TIGR00580 | mfd | DNA metabolism | transcription-repair coupling factor |
| TIGR00732 | dprA | Cellular processes | DNA protecting protein DprA |
| TIGR02386 | rpoC_TIGR | Transcription | DNA-directed RNA polymerase, beta' subunit |
| TIGR02692 | tRNA_CCA_actino | Protein synthesis | CCA tRNA nucleotidyltransferase |
| TIGR00459 | aspS_bact | Protein synthesis | aspartate--tRNA ligase |
| TIGR02504 | NrdJ_Z | Purines, pyrimidines, nucleosides, and nucleotides | ribonucleoside-diphosphate reductase, adenosylcobalamin-dependent |
| TIGR03594 | GTPase_EngA | Protein synthesis | ribosome-associated GTPase EngA |
| TIGR01137 | cysta_beta | Amino acid biosynthesis | cystathionine beta-synthase |
| TIGR00447 | pth | Protein synthesis | aminoacyl-tRNA hydrolase |
| TIGR03263 | guanyl_kin | Purines, pyrimidines, nucleosides, and nucleotides | guanylate kinase |
| TIGR00651 | pta | Energy metabolism | phosphate acetyltransferase |
| TIGR00187 | ribE | Biosynthesis of cofactors, prosthetic groups, and carriers | riboflavin synthase, alpha subunit |
| TIGR01455 | glmM | Central intermediary metabolism | phosphoglucosamine mutase |
| TIGR00343 | TIGR00343 | Biosynthesis of cofactors, prosthetic groups, and carriers | pyridoxal 5'-phosphate synthase, synthase subunit Pdx1 |
| TIGR00713 | hemL | Biosynthesis of cofactors, prosthetic groups, and carriers | glutamate-1-semialdehyde-2,1-aminomutase |
| TIGR00959 | ffh | Protein fate | signal recognition particle protein |
| TIGR00088 | trmD | Protein synthesis | tRNA (guanine(37)-N(1))-methyltransferase |
| TIGR01980 | sufB | Biosynthesis of cofactors, prosthetic groups, and carriers | FeS assembly protein SufB |
| TIGR00615 | recR | DNA metabolism | recombination protein RecR |
| TIGR00263 | trpB | Amino acid biosynthesis | tryptophan synthase, beta subunit |
| TIGR02075 | pyrH_bact | Purines, pyrimidines, nucleosides, and nucleotides | UMP kinase |
| TIGR01039 | atpD | Energy metabolism | ATP synthase F1, beta subunit |
| TIGR01520 | FruBisAldo_II_A | Energy metabolism | fructose-bisphosphate aldolase, class II |
| TIGR00184 | purA | Purines, pyrimidines, nucleosides, and nucleotides | adenylosuccinate synthase |
| TIGR01302 | IMP_dehydrog | Purines, pyrimidines, nucleosides, and nucleotides | inosine-5'-monophosphate dehydrogenase |
| TIGR00670 | asp_carb_tr | Purines, pyrimidines, nucleosides, and nucleotides | aspartate carbamoyltransferase |
| TIGR01050 | rpsS_bact | Protein synthesis | ribosomal protein uS19 |
| TIGR01011 | rpsB_bact | Protein synthesis | ribosomal protein uS2 |

# Table S2 – Bacterial strains used in this study.

| **Species/strain** | **Description** | **Source** | **Closest NCBI Refseq Genome** | **NCBI Refseq Assembly** | **Average Nucleotide Identity** |
| --- | --- | --- | --- | --- | --- |
| *Streptomyces sp.* strain A | Environmental strains isolated from Cedar Creek Ecosystem Science Reserve in Minnesota, USA. | ^2^ | *Streptomyces virginiae* | GCF_000716685 | 98.14 |
| *Streptomyces sp.* strain B |  |  | *Streptomyces hygroscopicus* | GCF_026168105 | 86.59 |
| *Streptomyces sp.* strain C |  |  | *Streptomyces griseochromogenes* | GCF_001542625 | 91.72 |
| *Streptomyces sp.* strain D |  |  | *Streptomyces hokutonensis* | GCF_000376565 | 96.38 |
| *Streptomyces sp.* strain C *desD* Q140* | Strain C with mutation to disrupt desferrioxamine biosynthesis | This work | *Streptomyces griseochromogenes* | GCF_001542625 | 91.72 |
| *Streptomyces sp.* strain C *desD* W241* | Strain C with mutation to disrupt desferrioxamine biosynthesis | This work | *Streptomyces griseochromogenes* | GCF_001542625 | 91.72 |
| *Escherichia coli* ET12567 [pUZ8002] | Conjugation donor strain | ^3^ | - |  | - |

# Table S3 – Plasmids used in this study, with Addgene repository numbers.

| **Plasmids** | **Description** | **Addgene Number** | **Source** |
| --- | --- | --- | --- |
| pCRISPR-cBEST | *Streptomyces* base editing plasmid | 125689 | ^4^ |
| pTE1643 | For generating mutation W241* in DesD | 219650 | This work |
| pTE1644 | For generating mutation Q140* in DesD | 219651 | This work |

# Table S4 – Oligonucleotides used in this study.

| **Primer Name** | **Sequence (5’ to 3’)** | **Description** |
| --- | --- | --- |
| oJC134 | CGGTTGGTAGGATCGACGGCTGTTCCACCA CTGCCAGGGGGTTTTAGAGCTAGAAATAGC | Insertion of W241* to pCRISPR-cBEST by single stranded NEB HiFi Assembly |
| oJC135 | CGGTTGGTAGGATCGACGGCGGCTTCCAGG CGATCGAGACGTTTTAGAGCTAGAAATAGC | Insertion of Q140* to pCRISPR-cBEST by single stranded NEB HiFi Assembly |
| sgRNA-test-F | AATTGTACGCGGTCGATCTT | Sequencing of protospacers in plasmids |
| sgRNA-test-R | TACGTAAAAAAAGCACCGAC |  |
| oJC136 | TGTGGCTGAGCGACGAGA | PCR amplification and Sanger sequencing of *desD* mutation in the chromosome |
| oJC137 | GGTGTTGAAGAAGGTCCGTATG |  |

# Table S5 – AntiSMASH summary output of predicted BGC of Strain A

| **Region** |  | **Type** | **From** | **To** | **Most similar known cluster** | | | **Similarity** | |
| --- | --- | --- | --- | --- | --- | --- | --- | --- | --- |
| Region 1.1 |  | thiopeptide,LAP | 40,628 | 70,694 | lactazole | RiPP:Thiopeptide | 55% | |  |
| Region 1.2 |  | ectoine | 98,189 | 108,207 | kosinostatin | NRP+Polyketide | 13% | |  |
| Region 1.3 |  | butyrolactone | 136,357 | 145,537 | lactonamycin | Polyketide | 7% | |  |
| Region 1.4 |  | terpene | 165,121 | 183,576 | avermitilol | Terpene | 100% | |  |
| Region 1.5 |  | ectoine | 646,027 | 656,431 | ectoine | Other | 100% | |  |
| Region 1.6 |  | T2PKS | 719,087 | 791,617 | spore pigment | Polyketide | 66% | |  |
| Region 1.7 |  | NRPS | 1,217,755 | 1,282,978 | JBIR-126 | NRP | 96% | |  |
| Region 1.8 |  | NRPS,T1PKS | 1,348,968 | 1,412,661 | coelichelin | NRP | 72% | |  |
| Region 1.9 |  | butyrolactone | 1,444,453 | 1,453,838 | Neocarzinostatin | Polyketide:Iterative type I polyketide+Polyketide:Enediyne type I polyketide | 6% | |  |
| Region 1.10 |  | betalactone,NRPS,T1PKS | 1,483,068 | 1,544,952 | pyrroloformamide A/pyrroloformamide B/  pyrroloformamide D/pyrroloformamide C | NRP+Other | 8% | |  |
| Region 1.11 |  | NI-siderophore | 3,096,914 | 3,107,836 | desferrioxamin B | Other | 100% | |  |
| Region 1.12 |  | ladderane,phosphonate,T1PKS,  nucleoside | 3,528,517 | 3,597,786 | dehydrofosmidomycin | Other | 23% | |  |
| Region 1.13 |  | butyrolactone | 4,231,389 | 4,242,402 | alanylclavam/2-hydroxymethylclavam/  2-formyloxymethylclavam/clavam-2-carboxylate | Other:Non-NRP beta-lactam | 12% | |  |
| Region 1.14 |  | arylpolyene,lanthipeptide-class-iii | 5,098,563 | 5,139,726 | atratumycin | NRP | 10% | |  |
| Region 1.15 |  | NI-siderophore | 6,031,856 | 6,045,298 |  | | | | |
| Region 1.16 |  | NRPS,T1PKS,other | 6,137,706 | 6,243,541 | himastatin | NRP | 52% | |  |
| Region 1.17 |  | terpene | 6,468,249 | 6,489,909 | geosmin | Terpene | 100% | |  |
| Region 1.18 |  | T1PKS | 6,493,588 | 6,535,051 | 4-hexadecanoyl-3-hydroxy-2-(hydroxymethyl)  -2H-furan-5-one | Polyketide | 45% | |  |
| Region 1.19 |  | T1PKS | 6,554,088 | 6,598,466 | crocacin | NRP+Polyketide | 9% | |  |
| Region 1.20 |  | terpene | 6,878,944 | 6,905,827 | hopene | Terpene | 61% | |  |
| Region 1.21 |  | T1PKS,hglE-KS | 6,963,138 | 7,014,751 | hexacosalactone A | Other | 9% | |  |
| Region 1.22 |  | NRPS | 7,054,956 | 7,140,587 | leupeptin Pr/leupeptin Ac | NRP | 100% | |  |
| Region 1.23 |  | NRPS | 7,240,291 | 7,285,345 | friulimicin A/friulimicin B/friulimicin C/friulimicin D | NRP | 12% | |  |
| Region 1.24 |  | terpene | 7,487,169 | 7,506,809 | bombyxamycin A/bombyxamycin B | Polyketide | 3% | |  |
| Region 1.25 |  | lanthipeptide-class-iii | 7,576,410 | 7,599,118 | SapB | RiPP:Lanthipeptide | 100% | |  |
| Region 1.26 |  | terpene | 7,637,239 | 7,657,906 | ebelactone | Polyketide | 5% | |  |
| Region 1.27 |  | terpene | 7,741,551 | 7,762,456 | monensin | Polyketide | 5% | |  |
| Region 1.28 |  | melanin | 7,766,398 | 7,793,205 | istamycin | Saccharide | 4% | |  |
| Region 1.29 |  | NI-siderophore | 7,922,498 | 7,935,689 |  | | | | |
| Region 1.30 |  | T3PKS | 7,965,814 | 8,006,875 | alkylresorcinol | Polyketide | 100% | |  |
| Region 1.31 |  | CDPS | 8,054,240 | 8,074,980 |  | | | | |
| Region 1.32 |  | NAPAA | 8,076,720 | 8,110,631 | ε-Poly-L-lysine | NRP | 100% | |  |
| Region 1.33 |  | NRPS | 8,180,585 | 8,223,761 | antipain | NRP | 100% | |  |
| Region 2.1 |  | lanthipeptide-class-ii | 275,029 | 297,872 |  | | | | |
| Region 2.2 |  | NRPS | 323,365 | 368,449 | mycotrienin I | NRP+Polyketide | 7% | |  |
| Region 2.3 |  | terpene | 484,903 | 505,997 | goadsporin | RiPP:LAP | 12% | |  |
| Region 3.1 |  | terpene | 45,362 | 66,456 | hopene | Terpene | 15% | |  |

# Table S6 – AntiSMASH summary output of predicted biosynthetic gene clusters of Strain B

| **Region** | **Type** | **From** | **To** | **Most similar known cluster** | | **Similarity** |
| --- | --- | --- | --- | --- | --- | --- |
| Region 1 | NRPS,NAPAA | 668,339 | 711,608 | stenothricin | NRP:Cyclic depsipeptide | 18% |
| Region 2 | melanin | 780,568 | 790,951 | melanin | Other | 42% |
| Region 3 | T3PKS | 1,733,998 | 1,772,465 | naringenin | Polyketide:Type III polyketide | 100% |
| Region 4 | NAPAA | 2,316,007 | 2,346,449 | chalcomycin A | Polyketide | 9% |
| Region 5 | ectoine | 2,483,438 | 2,493,836 | ectoine | Other | 100% |
| Region 6 | NI-siderophore | 3,670,589 | 3,681,869 | desferrioxamin B/ desferrioxamine E | Other | 66% |
| Region 7 | T2PKS | 4,516,705 | 4,587,123 | spore pigment | Polyketide | 83% |
| Region 8 | terpene | 6,234,626 | 6,255,590 | albaflavenone | Terpene | 100% |
| Region 9 | NI-siderophore | 6,908,517 | 6,918,649 |  |  |  |
| Region 10 | terpene | 7,199,284 | 7,220,754 | geosmin | Terpene | 100% |
| Region 11 | NI-siderophore | 7,338,959 | 7,352,054 | grincamycin | Polyketide:Type II polyketide+Saccharide:Hybrid/tailoring saccharide | 8% |
| Region 12 | T3PKS,T2PKS, oligosaccharide | 7,734,334 | 7,841,211 | galtamycin C/galtamycin D | Polyketide | 97% |
| Region 13 | terpene | 8,178,453 | 8,205,183 | hopene | Terpene | 92% |
| Region 14 | NAPAA | 8,374,245 | 8,408,129 | ε-Poly-L-lysine | NRP | 100% |

# Table S7 – AntiSMASH summary output of predicted biosynthetic gene clusters of Strain C

| **Region** | **Type** | **From** | **To** | **Most similar known cluster** | | **Similarity** |
| --- | --- | --- | --- | --- | --- | --- |
| Region 1.1 | terpene | 47,498 | 68,199 |  |  |  |
| Region 1.2 | lanthipeptide-class-iv,T1PKS | 163,335 | 227,178 | blasticidin S | Other | 67% |
| Region 1.3 | lanthipeptide-class-ii,T1PKS,NRPS,NAPAA | 264,537 | 389,158 | meilingmycin | Polyketide | 14% |
| Region 1.4 | T1PKS | 417,704 | 460,207 | fuelimycin A/fuelimicin B/fuelimicin C | Polyketide | 100% |
| Region 1.5 | lanthipeptide-class-iii | 630,789 | 653,479 | informatipeptin | RiPP:Lanthipeptide | 100% |
| Region 1.6 | terpene | 1,211,039 | 1,237,106 | hopene | Terpene | 76% |
| Region 1.7 | NRPS,terpene | 1,364,866 | 1,500,806 | enduracidin | NRP | 20% |
| Region 1.8 | redox-cofactor,NRP-metallophore,NRPS | 1,560,190 | 1,617,690 | aminochelin/azotochelin/protochelin | NRP | 50% |
| Region 1.9 | T1PKS,NRPS | 1,715,607 | 1,802,468 | guadinomine/guadinomine B/guadinomic acid | NRP+Polyketide | 30% |
| Region 1.10 | NI-siderophore | 1,843,746 | 1,855,703 | grincamycin | Polyketide:Type II polyketide+Saccharide:Hybrid/tailoring saccharide | 8% |
| Region 1.11 | terpene | 2,048,497 | 2,069,839 | geosmin | Terpene | 100% |
| Region 1.12 | T1PKS,other | 2,173,620 | 2,225,069 | tambjamine BE-18591 | Alkaloid+Polyketide | 21% |
| Region 1.13 | NRPS,other | 2,242,349 | 2,297,177 | leucomycin | Polyketide | 11% |
| Region 1.14 | NRPS,T1PKS,NI-siderophore | 2,375,270 | 2,550,843 | apoptolidin | Polyketide | 33% |
| Region 1.15 | transAT-PKS,NRPS | 2,712,367 | 2,803,794 | phthoxazolin | NRP+Polyketide | 20% |
| Region 1.16 | T1PKS | 3,126,384 | 3,254,278 | oligomycin | Polyketide | 88% |
| Region 1.17 | terpene | 3,377,452 | 3,398,257 | albaflavenone | Terpene | 100% |
| Region 1.18 | T1PKS,aminocoumarin | 3,580,933 | 3,667,264 | rubradirin | Polyketide | 37% |
| Region 1.19 | lanthipeptide-class-v | 4,996,191 | 5,040,202 | TVA-YJ-2 | RiPP | 33% |
| Region 1.20 | T2PKS | 5,161,391 | 5,233,915 | spore pigment | Polyketide | 83% |
| Region 1.21 | LAP | 5,294,935 | 5,315,837 |  |  |  |
| Region 1.22 | NRPS | 5,400,146 | 5,467,893 | cadaside A/cadaside B | NRP | 23% |
| Region 1.23 | NI-siderophore | 6,009,640 | 6,020,097 | desferrioxamin B/desferrioxamine E | Other | 83% |
| Region 1.24 | thiopeptide,LAP | 6,053,776 | 6,083,697 | lactazole | RiPP:Thiopeptide | 66% |
| Region 1.25 | melanin | 6,119,718 | 6,130,024 | melanin | Other | 100% |
| Region 1.26 | terpene | 6,567,692 | 6,587,879 | kanamycin | Saccharide | 12% |
| Region 1.27 | ectoine | 7,221,143 | 7,231,547 | ectoine | Other | 100% |
| Region 1.28 | NAPAA | 7,464,154 | 7,498,011 | ε-Poly-L-lysine | NRP | 100% |
| Region 1.29 | hglE-KS,T1PKS | 7,906,940 | 7,958,945 | hexacosalactone A | Other | 13% |
| Region 1.30 | T3PKS | 8,032,026 | 8,073,090 | flaviolin/1,3,6,8-tetrahydroxynaphthalene | Polyketide | 100% |
| Region 1.31 | NRPS,lassopeptide,prodigiosin | 8,075,489 | 8,127,578 | tyrobetaine | NRP | 80% |
| Region 1.32 | lassopeptide | 8,343,697 | 8,366,433 | lagmysin | RiPP | 80% |
| Region 1.33 | other | 8,445,212 | 8,486,585 | A-503083 A/A-503083 B/A-503083 E/A-503083 F | NRP | 7% |
| Region 1.34 | T1PKS | 8,588,568 | 8,632,532 | nocathiacin | RiPP:Thiopeptide | 4% |
| Region 1.35 | terpene,T1PKS | 8,751,862 | 8,824,417 | foxicin A/foxicin B/foxicin C/foxicin | NRP+Polyketide | 12% |
| Region 1.36 | terpene,melanin | 8,854,359 | 8,875,323 | melanin | Other | 57% |
| Region 1.37 | CDPS | 8,989,142 | 9,009,852 | prunipeptin | RiPP | 100% |
| Region 1.38 | terpene | 9,482,908 | 9,503,143 | pentalenolactone | Terpene | 58% |
| Region 1.39 | NI-siderophore | 9,736,740 | 9,750,061 | peucechelin | NRP | 10% |
| Region 1.40 | thioamitides | 9,881,411 | 9,903,805 |  |  |  |
| Region 1.41 | terpene | 9,965,234 | 9,986,130 | A54145 | NRP | 3% |

# Table S8 – AntiSMASH summary output of predicted biosynthetic gene clusters of Strain D

| **Region** | **Type** | **From** | **To** | **Most similar known cluster** | | **Similarity** |
| --- | --- | --- | --- | --- | --- | --- |
| Region 1.1 | lanthipeptide-class-i | 64,478 | 89,630 | griselimycin | NRP | 7% |
| Region 1.2 | NRPS,T1PKS | 576,859 | 632,927 | azicemicin B | Polyketide | 15% |
| Region 1.3 | NRPS | 809,297 | 861,939 | rimosamide | NRP | 35% |
| Region 1.4 | T3PKS | 1,898,605 | 1,939,669 | flaviolin/1,3,6,8-tetrahydroxynaphthalene | Polyketide | 100% |
| Region 1.5 | NRP-metallophore,NRPS,T1PKS | 2,050,581 | 2,225,915 | naphthomycin A | Polyketide | 71% |
| Region 1.6 | terpene | 2,579,103 | 2,596,731 | pradimicin-A | Polyketide | 7% |
| Region 1.7 | NAPAA | 2,661,295 | 2,694,387 | ε-Poly-L-lysine | NRP | 100% |
| Region 1.8 | ectoine | 2,973,183 | 2,983,587 | ectoine | Other | 100% |
| Region 1.9 | melanin | 4,149,041 | 4,159,484 | melanin | Other | 60% |
| Region 1.10 | NI-siderophore | 4,263,060 | 4,273,691 | desferrioxamin B/desferrioxamine E | Other | 83% |
| Region 1.11 | other | 4,368,015 | 4,408,635 | armeniaspirol A/armeniaspirol B/armeniaspirol C | Polyketide | 13% |
| Region 1.12 | T2PKS | 5,313,694 | 5,386,182 | α naphtocyclinoic acid/fogacin | Polyketide | 62% |
| Region 1.13 | terpene | 7,224,190 | 7,245,017 | albaflavenone | Terpene | 100% |
| Region 1.14 | NI-siderophore | 7,980,551 | 7,991,233 |  |  |  |
| Region 1.15 | betalactone | 8,198,105 | 8,230,139 |  |  |  |
| Region 1.16 | T1PKS,hglE-KS | 8,361,333 | 8,411,902 | hexacosalactone A | Other | 13% |
| Region 1.17 | terpene | 8,449,103 | 8,469,505 | geosmin | Terpene | 100% |
| Region 1.18 | NAPAA | 8,511,964 | 8,546,541 | stenothricin | NRP:Cyclic depsipeptide | 13% |
| Region 1.19 | butyrolactone | 8,648,889 | 8,659,815 |  |  |  |
| Region 1.20 | NI-siderophore | 8,714,937 | 8,728,045 | grincamycin | Polyketide:Type II polyketide+Saccharide:Hybrid/tailoring saccharide | 8% |
| Region 1.21 | terpene | 9,238,172 | 9,262,918 | hopene | Terpene | 92% |
| Region 1.22 | T1PKS | 9,417,317 | 9,458,319 | chlorothricin/deschlorothricin | Polyketide:Modular type I polyketide+Polyketide:Iterative type I polyketide+Saccharide:Oligosaccharide | 4% |
| Region 1.23 | terpene | 9,984,434 | 10,015,384 | ebelactone | Polyketide | 5% |
| Region 1.24 | T1PKS | 10,266,471 | 10,313,439 | desertomycin B/desertomycin A/desertomycin G | NRP | 10% |
| Region 1.25 | NRPS,nucleoside | 10,824,765 | 10,869,717 | toyocamycin | Other | 30% |

# Table S9 – Transcriptomic sample labelling and multiplexing. Samples were multiplexed into 4 mixes per replicate (n=3) per day (days 2, 3 and 4). Resulting in 36 multiplexed RNA-Seq samples.

| **Strain left** | **Mix number** | **Strain right** | **Mix number** | **Replicate number** |
| --- | --- | --- | --- | --- |
| A | 1 | A | 0 | 1 |
| A | 1 | A | 0 | 2 |
| A | 1 | A | 0 | 3 |
| A | 1 | A | 0 | 4 |
| C | 1 | C | 0 | 1 |
| C | 1 | C | 0 | 2 |
| C | 1 | C | 0 | 3 |
| C | 1 | C | 0 | 4 |
| D | 1 | D | 0 | 1 |
| D | 1 | D | 0 | 2 |
| D | 1 | D | 0 | 3 |
| D | 1 | D | 0 | 4 |
| B | 1 | B | 0 | 1 |
| B | 1 | B | 0 | 2 |
| B | 1 | B | 0 | 3 |
| B | 1 | B | 0 | 4 |
| A | 2 | C | 3 | 1 |
| A | 2 | C | 3 | 2 |
| A | 2 | C | 3 | 3 |
| A | 2 | C | 3 | 4 |
| C | 2 | B | 4 | 1 |
| C | 2 | B | 4 | 2 |
| C | 2 | B | 4 | 3 |
| C | 2 | B | 4 | 4 |
| C | 4 | D | 2 | 1 |
| C | 4 | D | 2 | 2 |
| C | 4 | D | 2 | 3 |
| C | 4 | D | 2 | 4 |
| A | 3 | B | 2 | 1 |
| A | 3 | B | 2 | 2 |
| A | 3 | B | 2 | 3 |
| A | 3 | B | 2 | 4 |
| B | 3 | D | 4 | 1 |
| B | 3 | D | 4 | 2 |
| B | 3 | D | 4 | 3 |
| B | 3 | D | 4 | 4 |
| A | 4 | D | 3 | 1 |
| A | 4 | D | 3 | 2 |
| A | 4 | D | 3 | 3 |
| A | 4 | D | 3 | 4 |


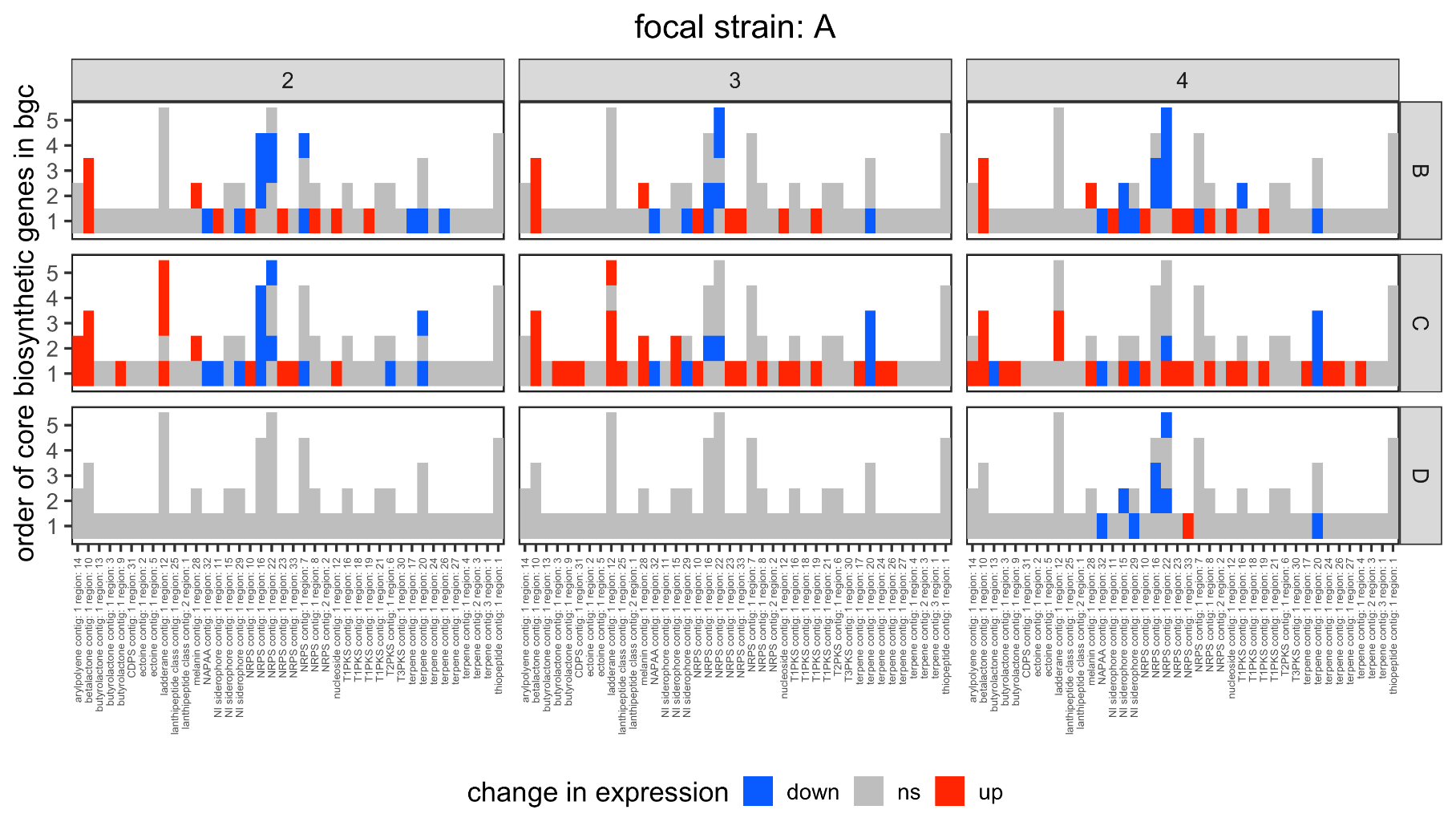


# Figure S1 – Significant changes in expression of strain A core BGC genes in response to partner strains (right label), relative to axenic cultures. Top labels correspond to sampling day 2, 3 or 4. Summary information on the corresponding BGCs is presented in Table S5.


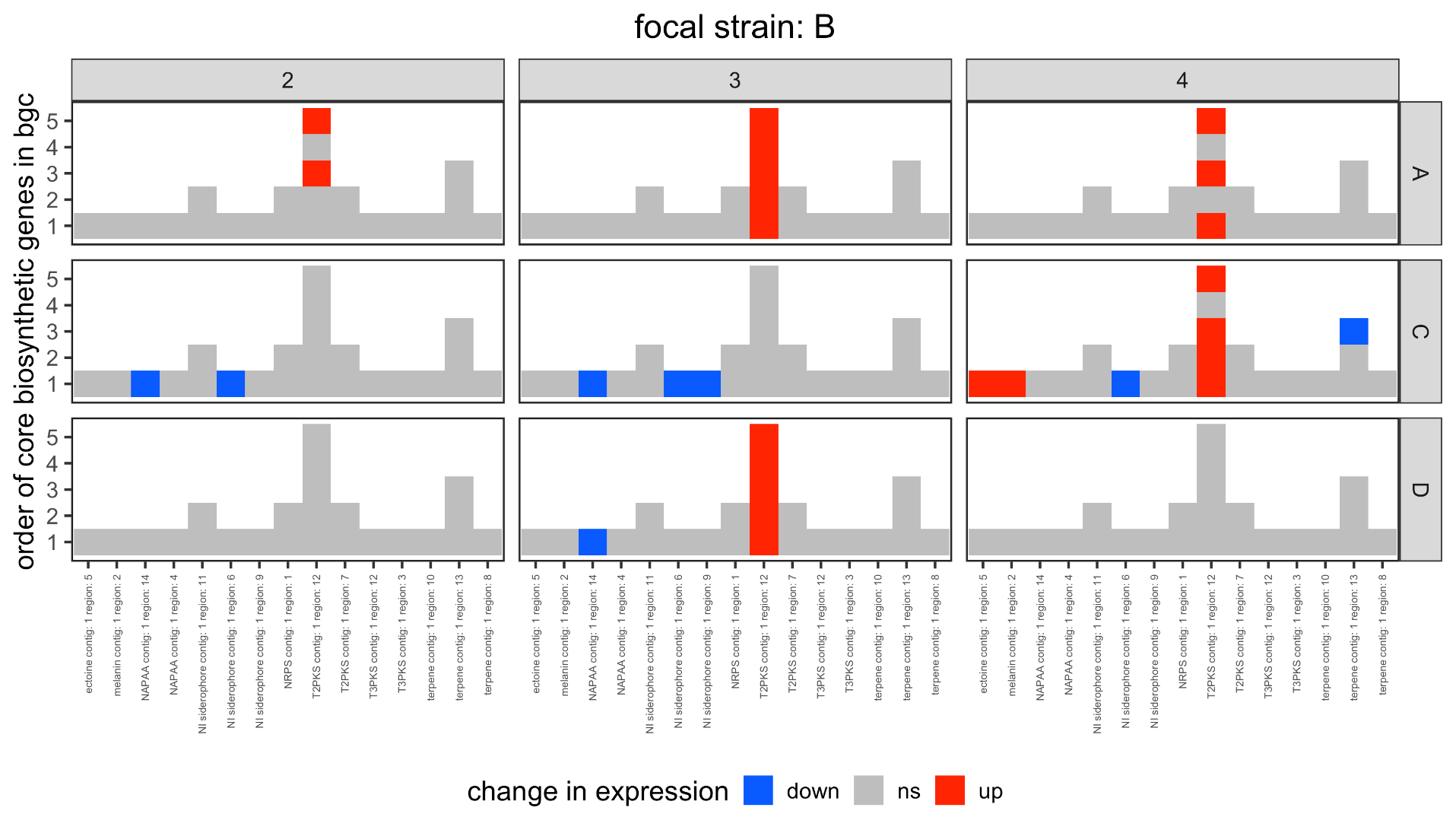


# Figure S2 – Significant changes in expression of strain B core BGC genes in response to partner strains (right label), relative to axenic cultures. Top labels correspond to sampling day 2, 3 or 4. Summary information on the corresponding BGCs is presented in Table S6.


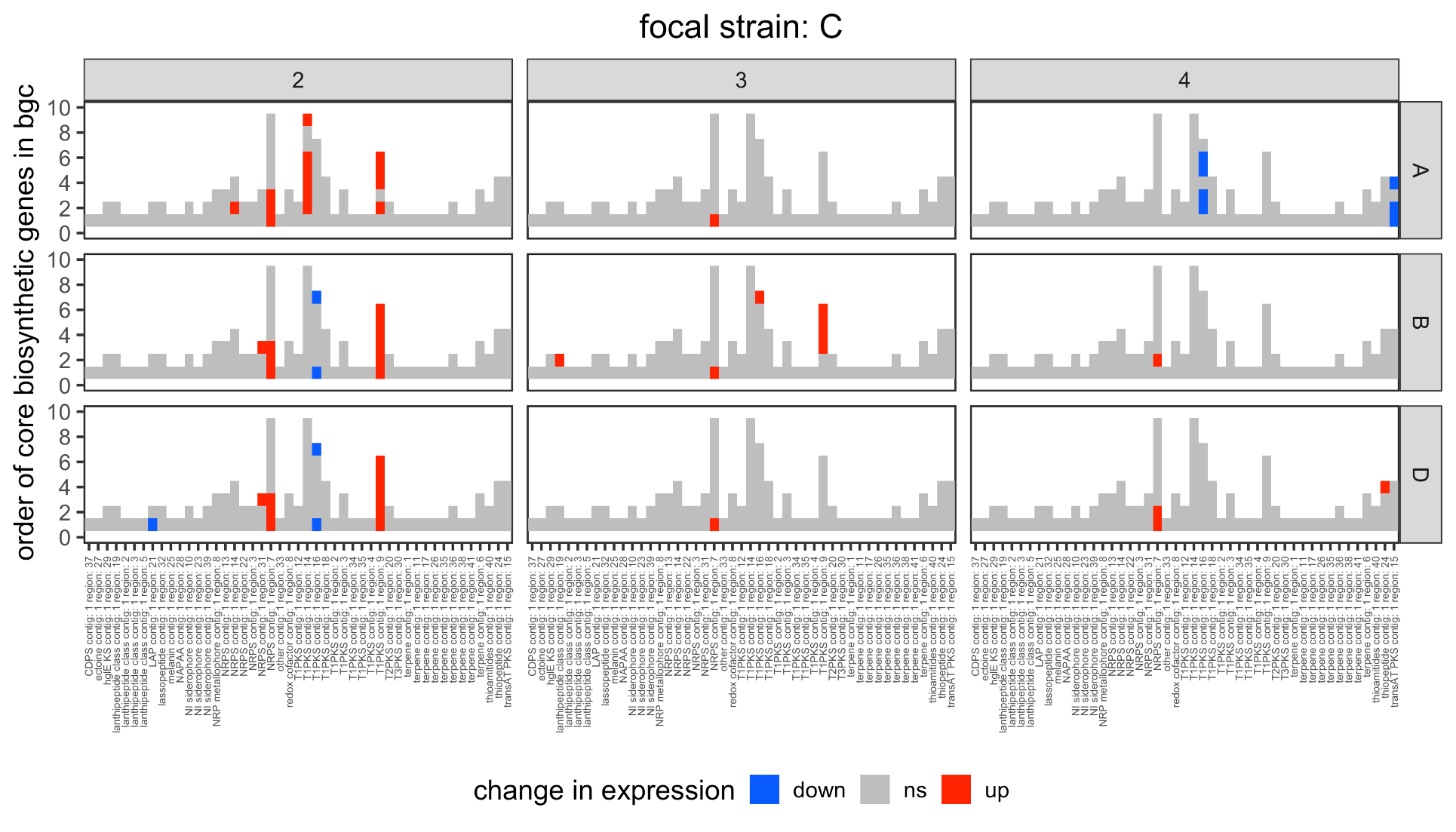


# Figure S3 – Significant changes in expression of strain C core BGC genes in response to partner strains (right label), relative to axenic cultures. Top labels correspond to sampling day 2, 3 or 4. Summary information on the corresponding BGCs is presented in Table S7.


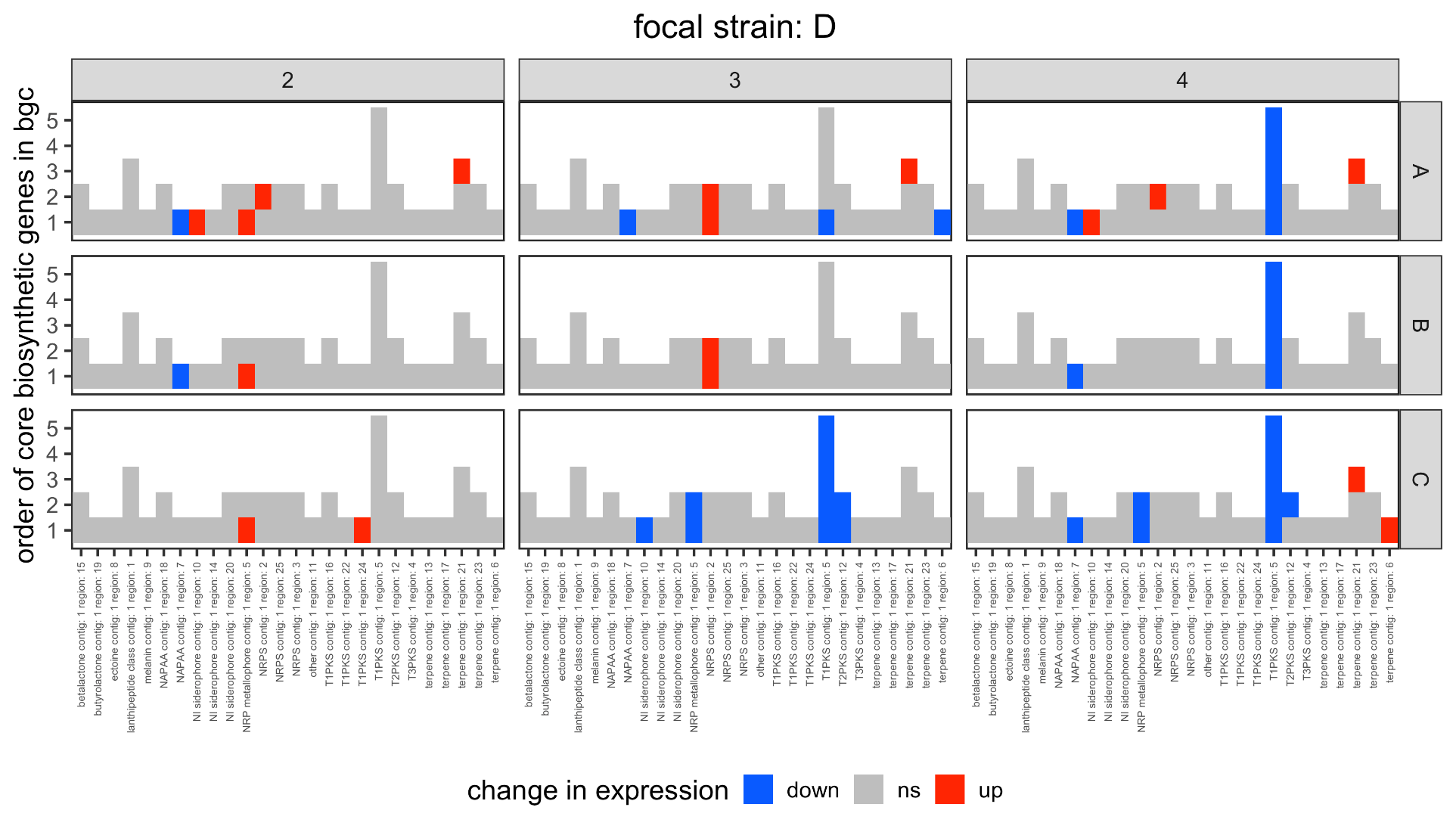


# Figure S4 – Significant changes in expression of strain D core BGC genes in response to partner strains (right label), relative to axenic cultures. Top labels correspond to sampling day 2, 3 or 4. Summary information on the corresponding BGCs is presented in Table S8.


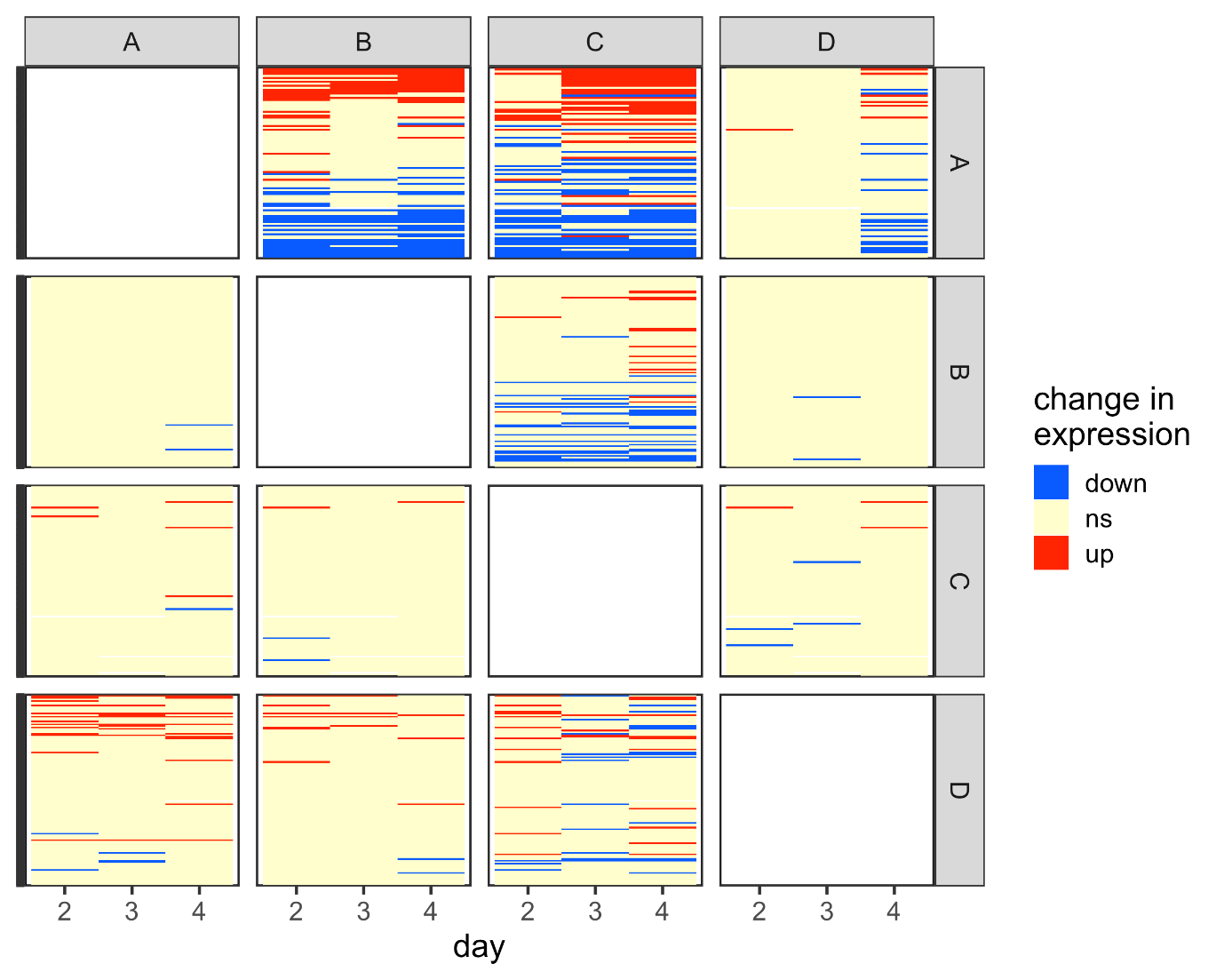


# Figure S5 – Heatmap of expression of iron-related genes filtered by annotation containing ‘ferr’, ‘iron’, ‘sidero’, ‘heme’, ‘haem’, ‘hemo’ and ‘fur’. Significantly (P<0.05) upregulated genes are indicated red, downregulated indicated blue.

**

# Figure S6 – Heatmaps of the normalised intensities associated with ions likely produced by the different strains. Ions detected in positive mode are on the left and negative mode on the right. Each column represents one sampling day, and each group of columns represent the partner strain. Putative compound annotations are included here, where ipaPy2 gives a posterior probability > 0.6.


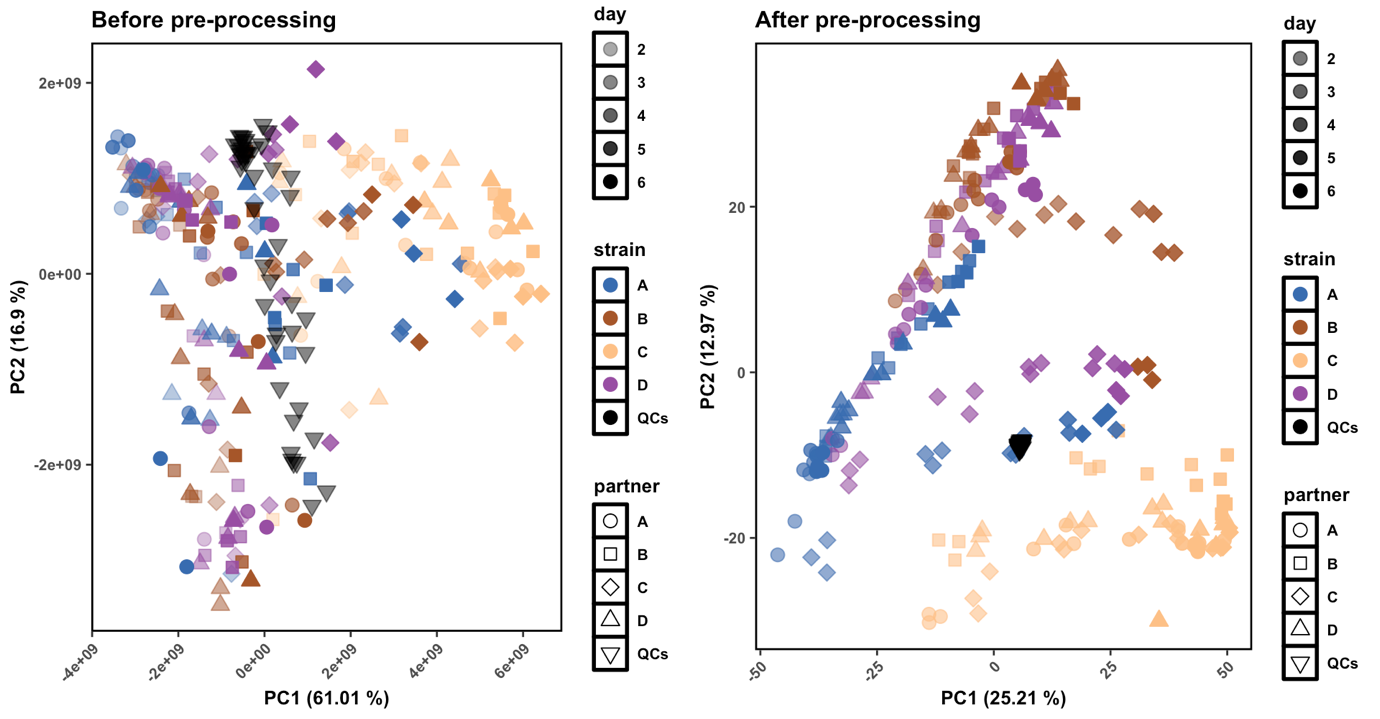
Figure S7 – Effect of QC Correction and Probabilistic Quotient Normalization (PQN) on Sample Clustering in Principal Component Analysis (PCA). Left Panel – PCA plot of the data before QC correction and PQN normalization. Samples exhibit wide dispersion, and QC samples (black diamonds) show considerable spread. Right Panel – PCA plot of the same data after QC correction and PQN normalization. The processing has resulted in a marked improvement in data structure. QC samples (black) are now tightly clustered, indicating a successful reduction in technical variability. Furthermore, biological groups show clearer separation and more distinct clustering.

#
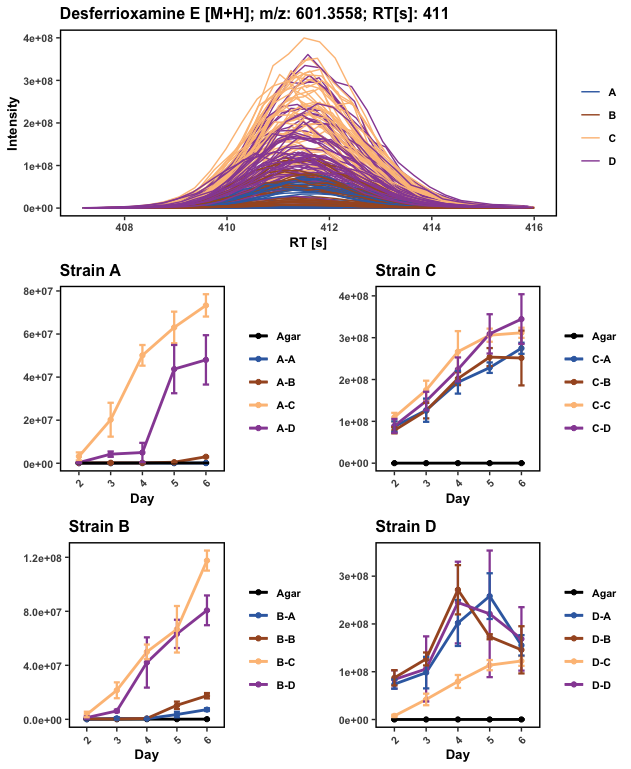
Figure S8 – LC-MS detection of DFO-E. Top panel overlays extracted ion chromatograms showing detected peak shape across the retention time window. Bottom panels show abundance of ion m/z 601.3558 corresponding to DFO-E [M+H] in each strain combination, including agar controls.


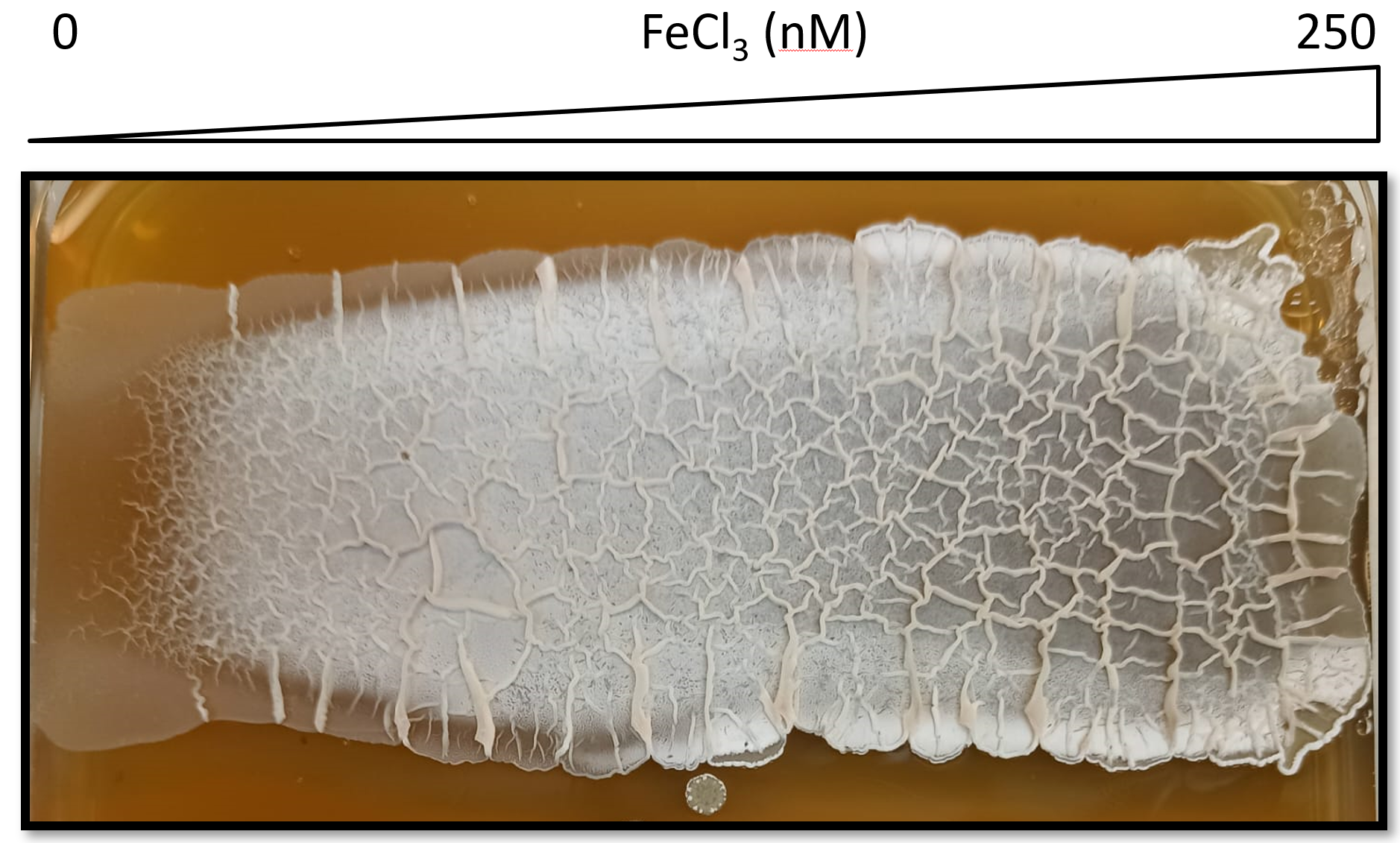


# Figure S9 – Response of Strain A to an iron chloride gradient. Strain A was incubated for 3 days at 30 °C on an ISP2 agar plate prepared with slanted layers of 0 and 250 nM FeCl_3_ to give a gradient approximately as indicated.


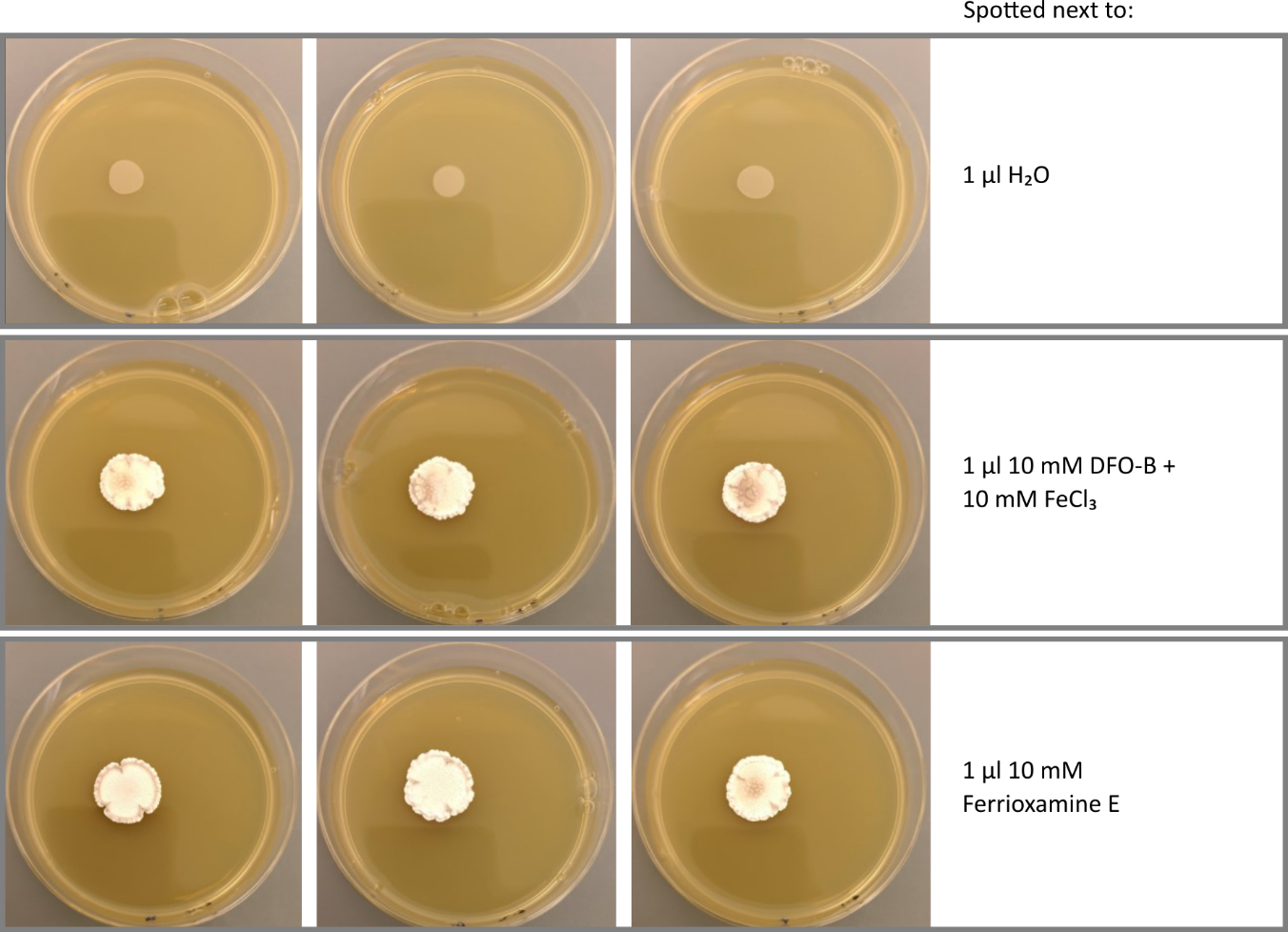


# Figure S10 – Strain A spotted 1 cm away from indicated compounds, after 4 days incubation at 30 °C, n=3.


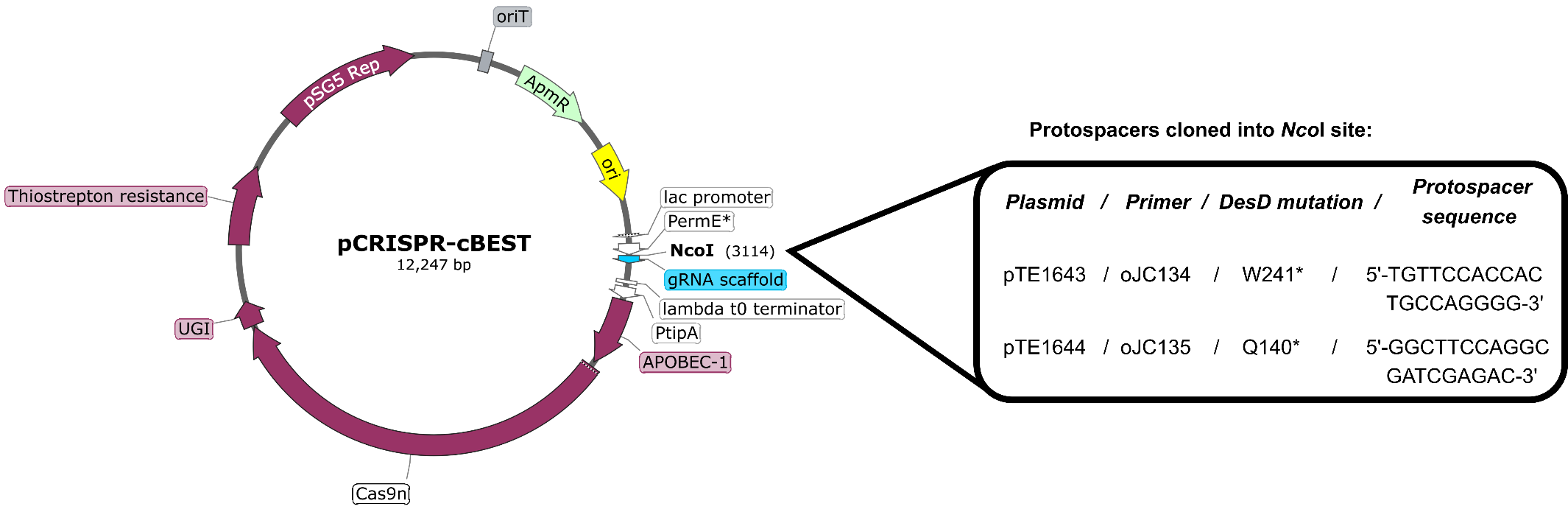


# Figure S11 – Overview of base editing CRISPR plasmid designs. Plasmids were constructed by NEBuilder Hifi Assembly into pCRISPR-cBEST linearised by *Nco*I digest and dephosphorylated with Quick CIP (NEB). Full oligonucleotide sequences are listed in Table S4.


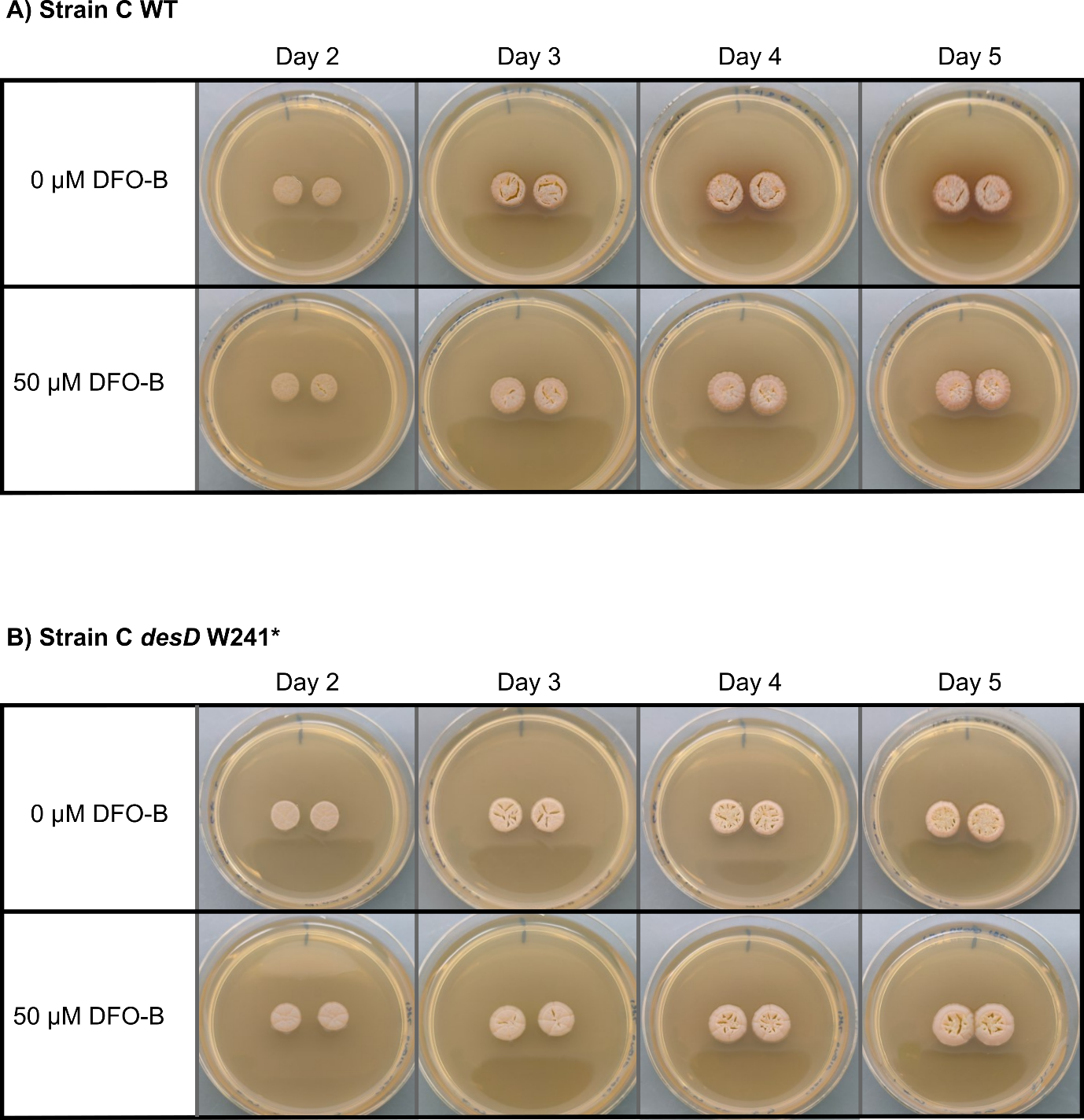


# Figure S12 – A) Daily phenotype photos of Strain C WT axenic pairs. The bottom row includes 50 µM DFO-B throughout the agar, the top row has none. B) Daily phenotype photos of Strain C *desD* W241* DFO-B biosynthesis mutants. The bottom row includes 50 µM DFO-B throughout the agar, the top row has none.


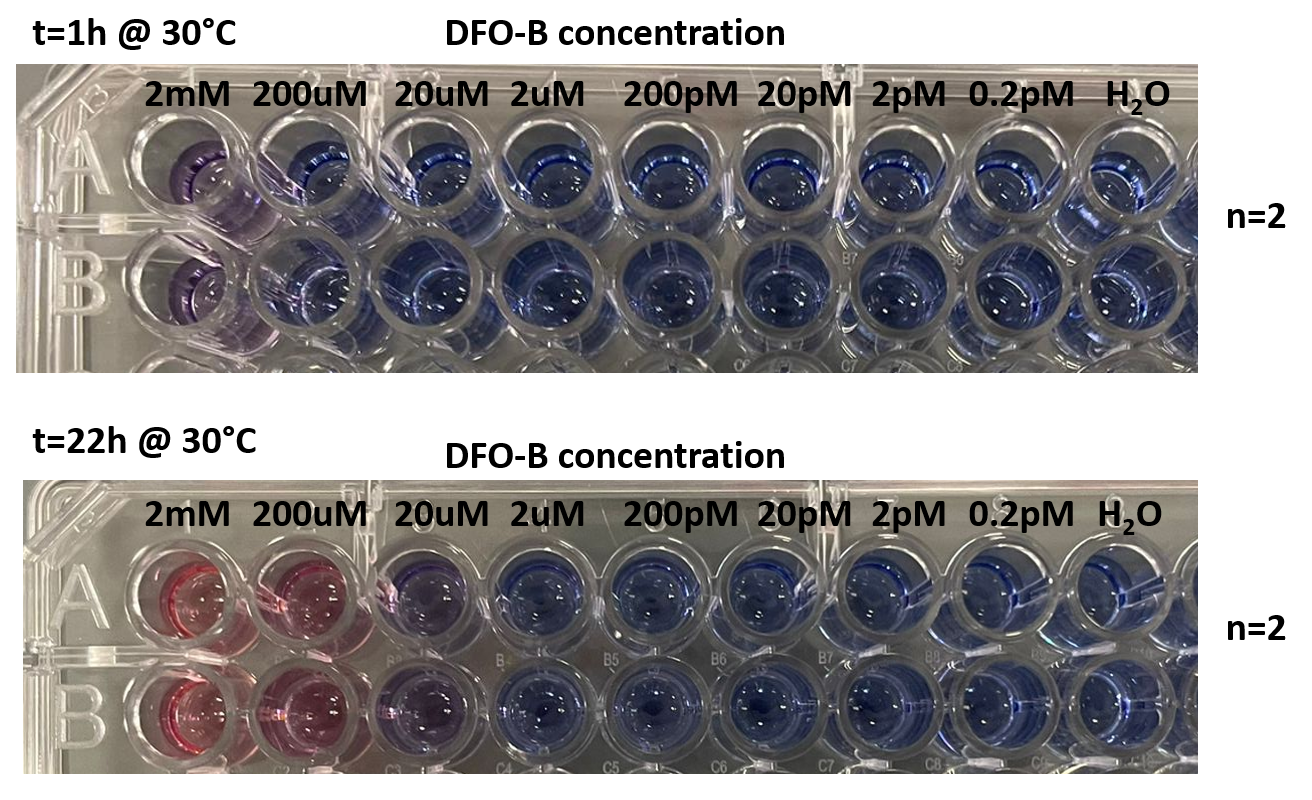


# Figure S13 – Chrome azurol solution control assay of DFO-B standards.


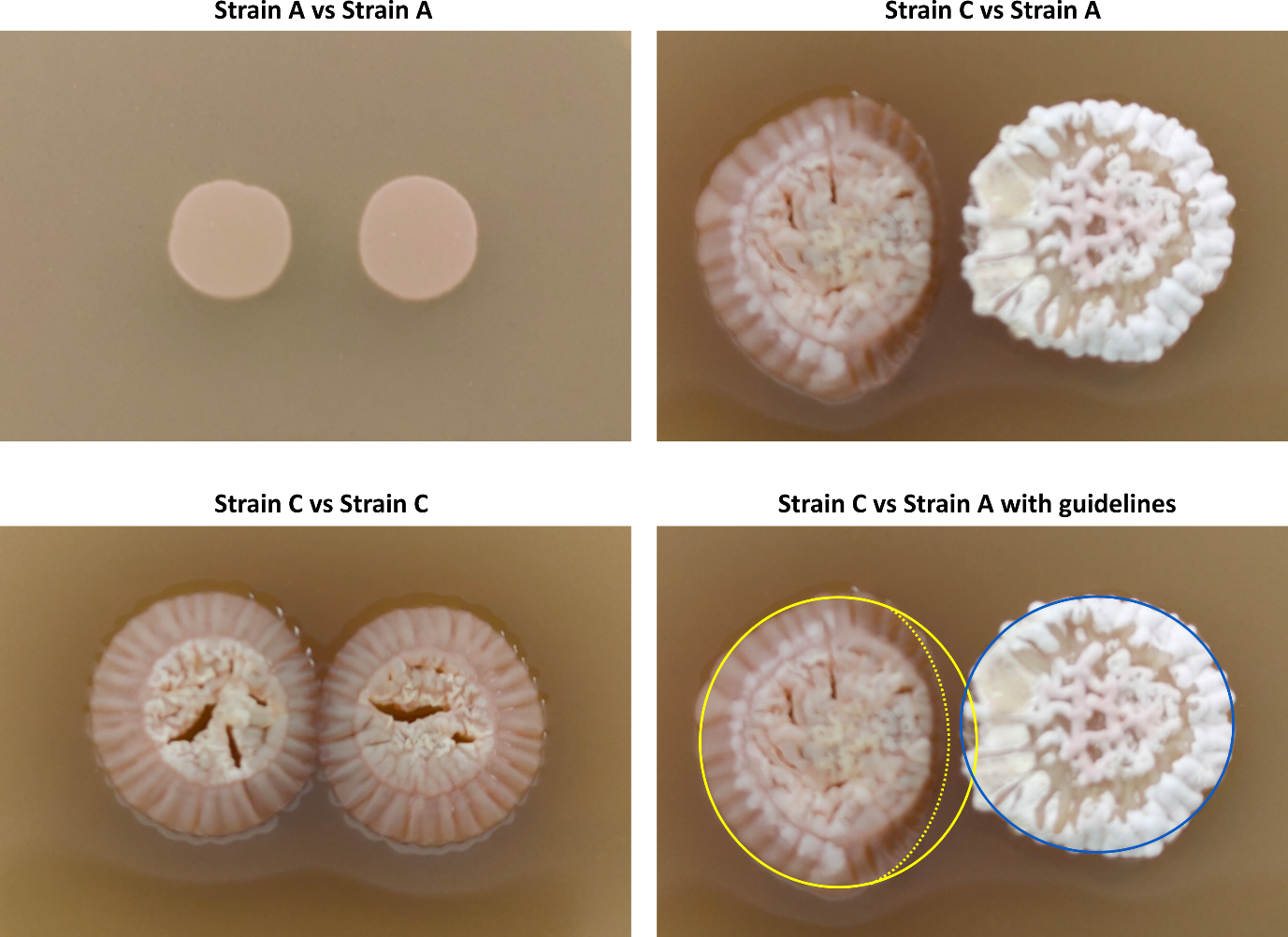


# Figure S14 – Zoomed photos of the interaction of strains A and C. Guidelines are added to help visualise the apparent directional inhibition of Strain C by Strain A, solid lines represent where a symmetrical colony would reach, dashed lines represent approximately where the colony border is. The top right photo is the same but unmarked.


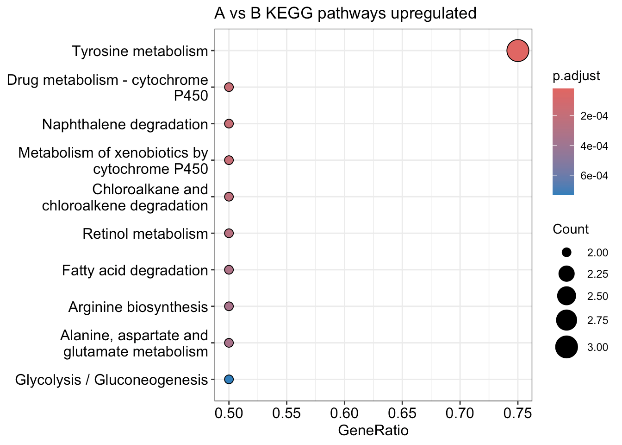

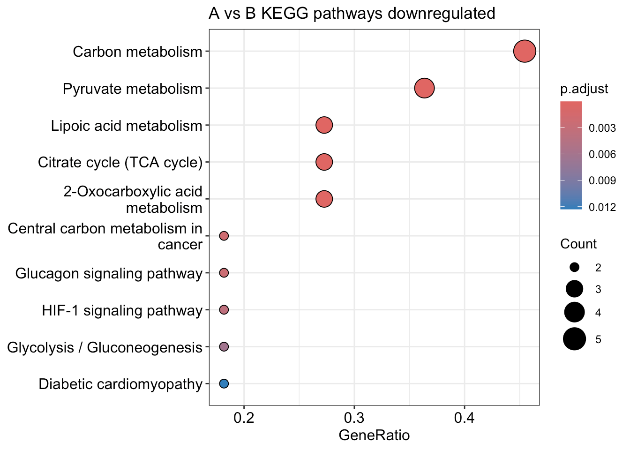


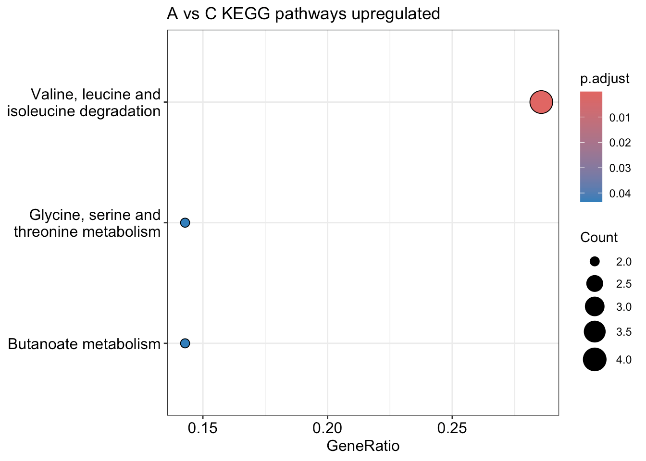

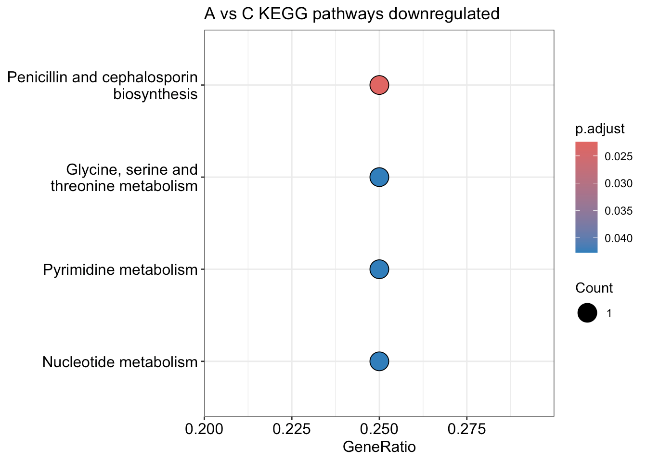


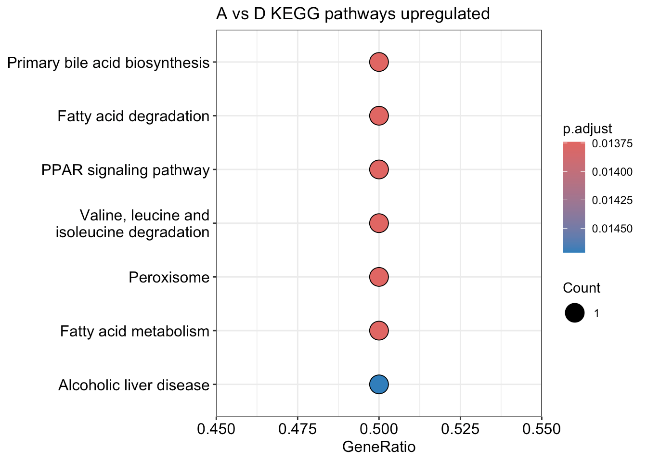

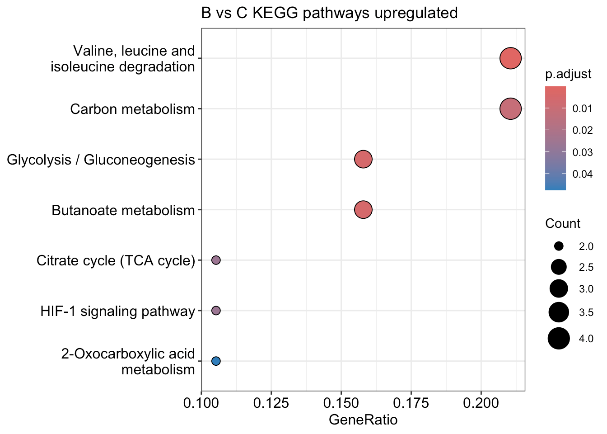

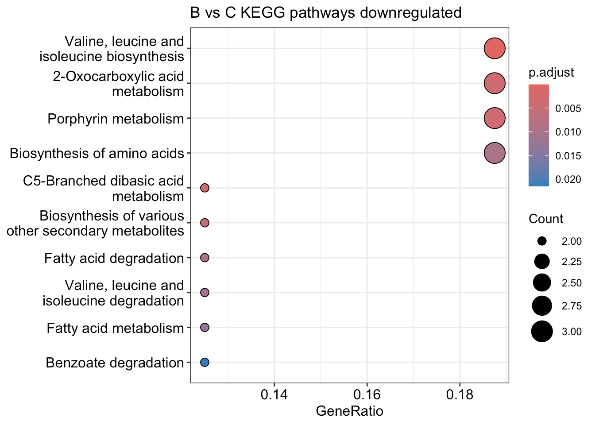

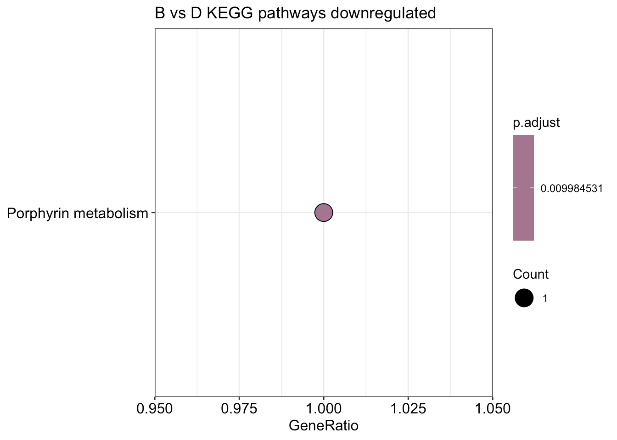


# Figure S15 – Enrichment analysis of **strain B** – KEGG pathways up/downregulated in the RNAseq data. Each panel shows pathways differentially expressed in strain B in the comparison given in the subtitle. For instance, the A vs D upregulated panel shows pathways upregulated in strain B when next to D compared with when next to A. Where comparisons are absent, e.g. A vs B, the analysis showed no pathways enriched.


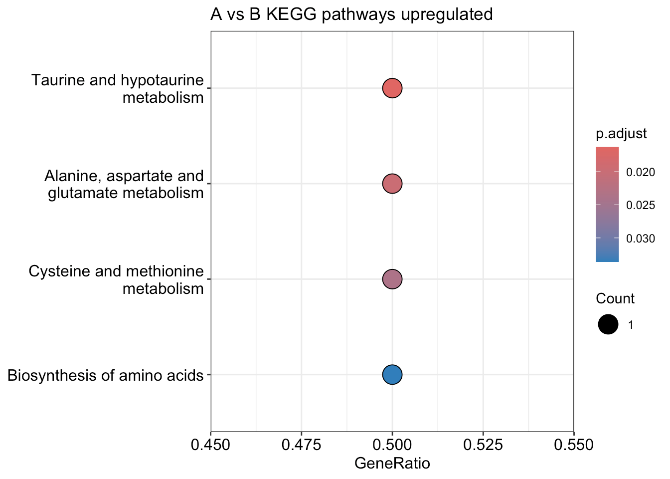

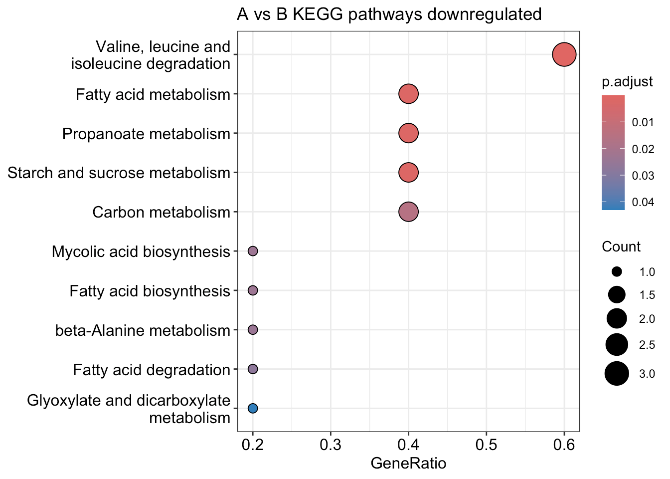

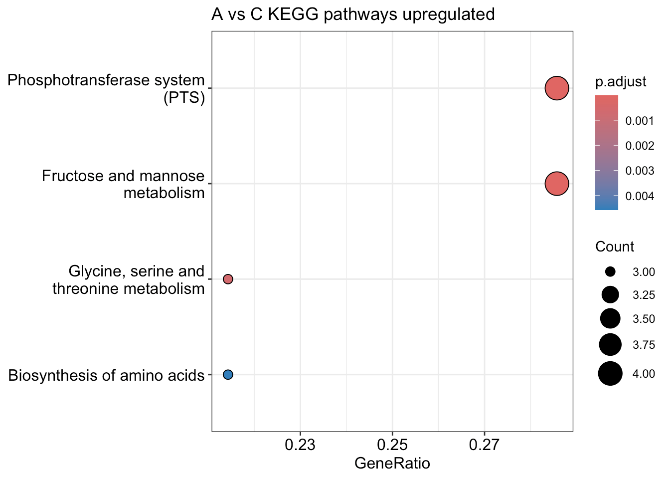

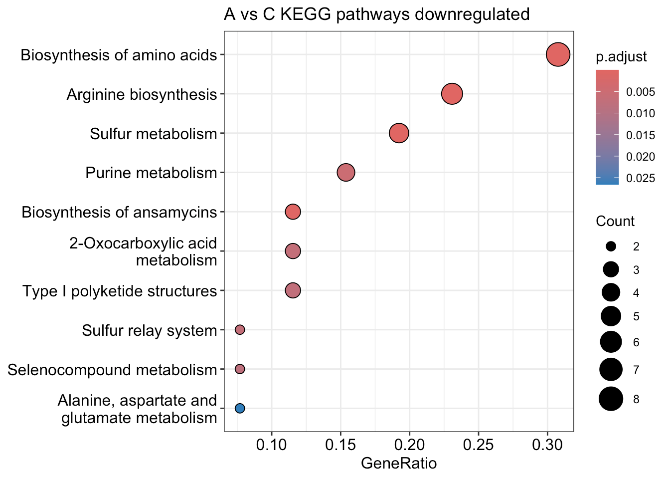

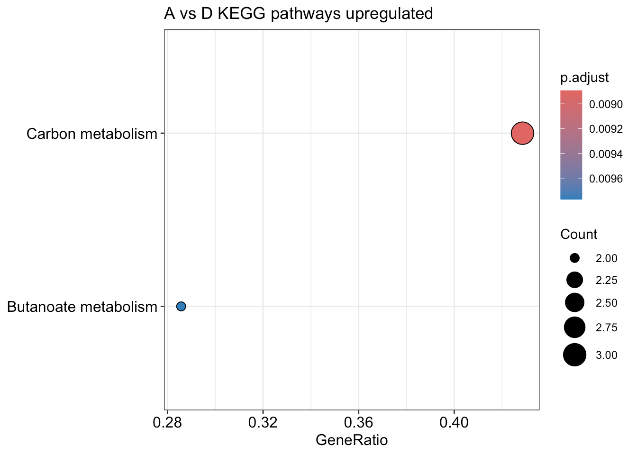

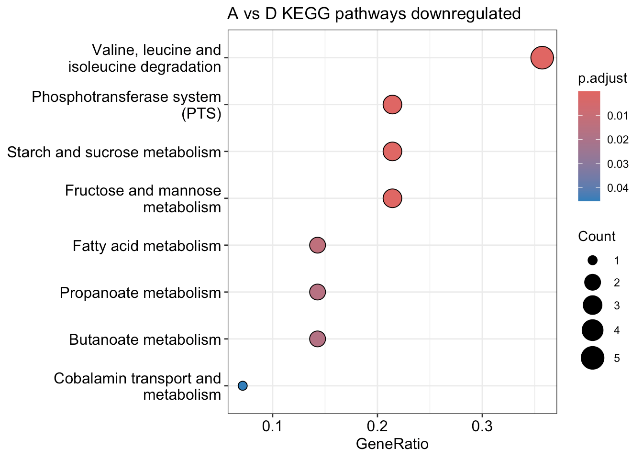


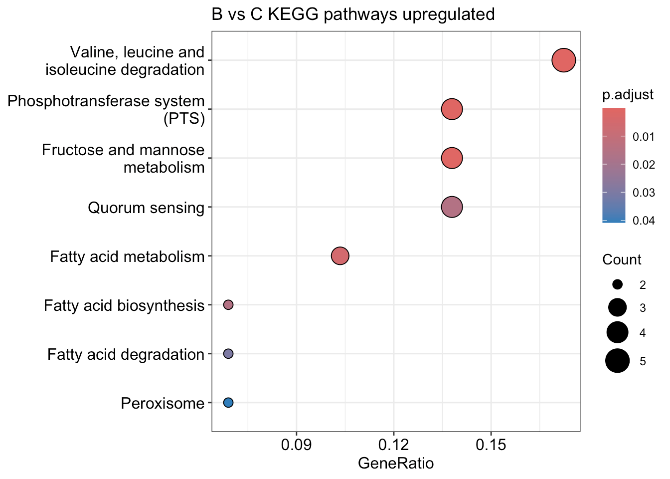

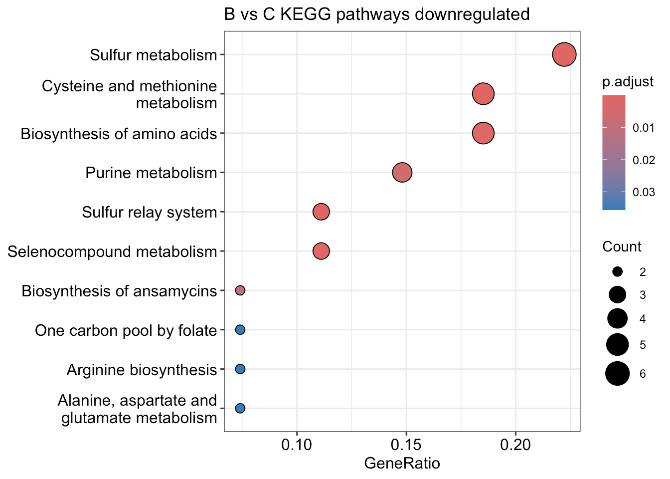


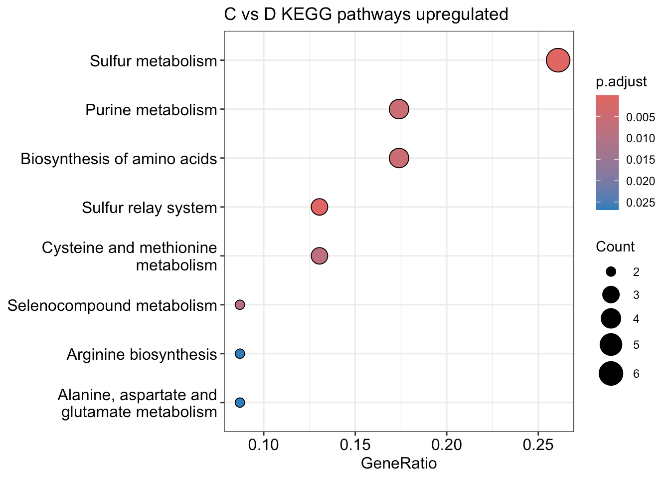

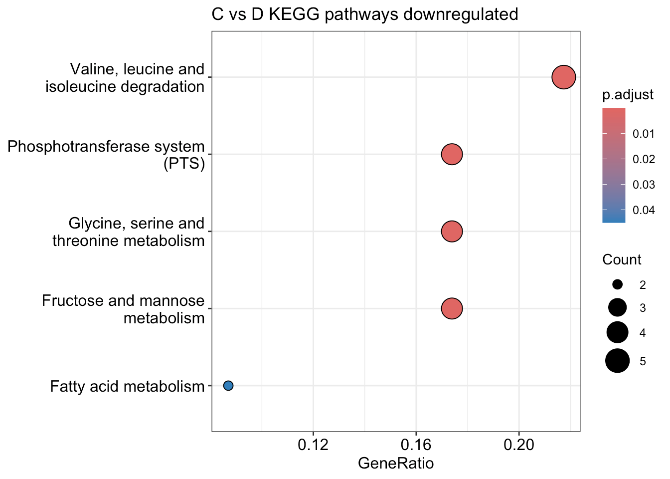


# Figure S16 – Enrichment analysis of **strain C** – KEGG pathways up/downregulated in the RNAseq data. Each panel shows pathways differentially expressed in strain C in the comparison given in the subtitle. For instance, the A vs D upregulated panel shows pathways upregulated in strain C when next to D compared with when next to A. Where comparisons are absent, e.g. B vs D, the analysis showed no pathways enriched.


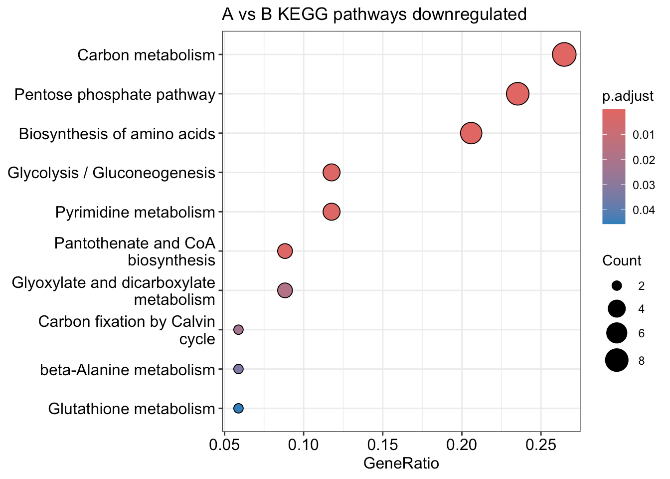

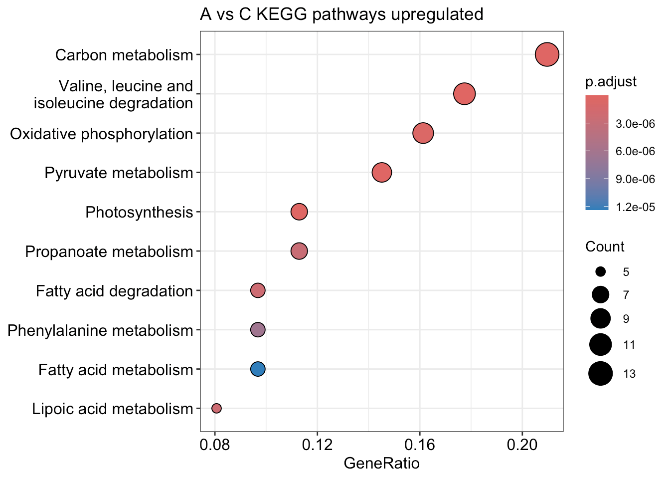


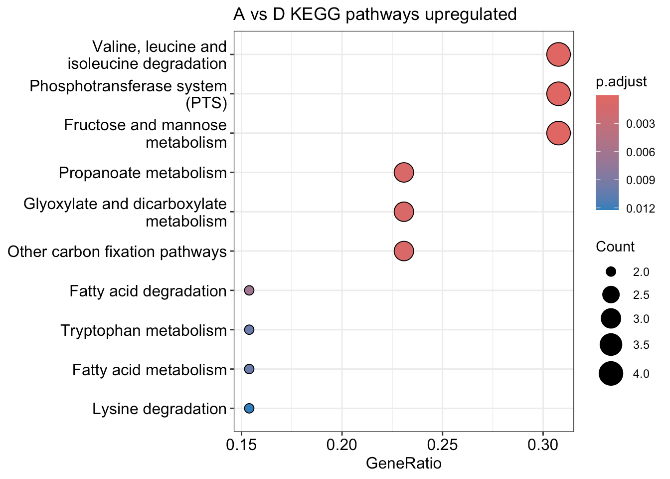

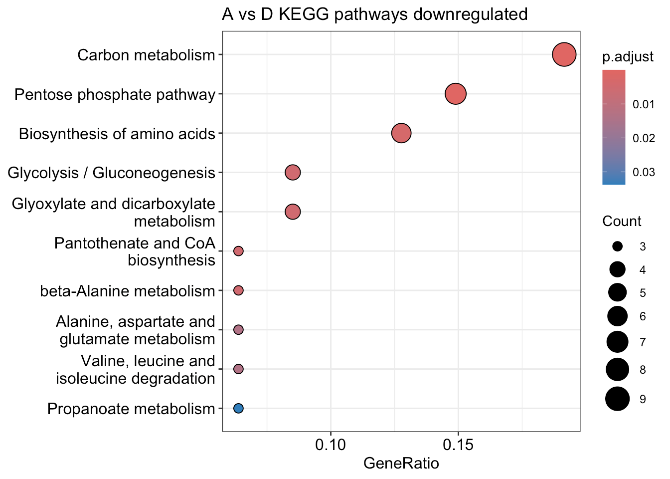

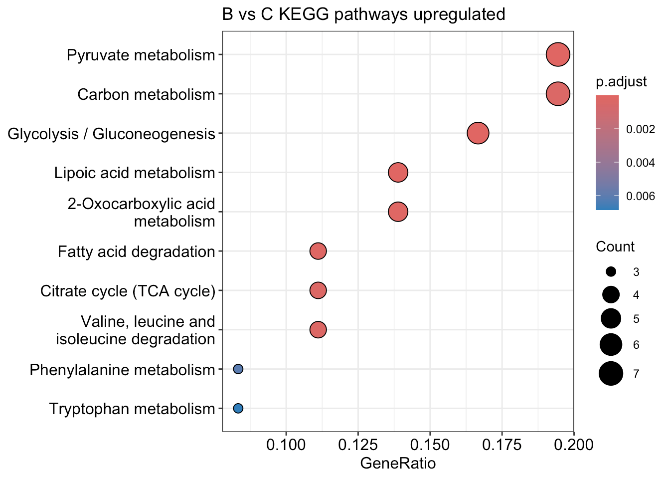

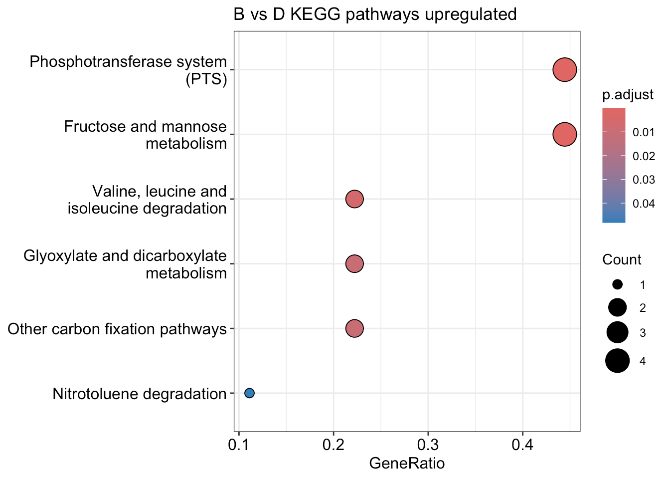


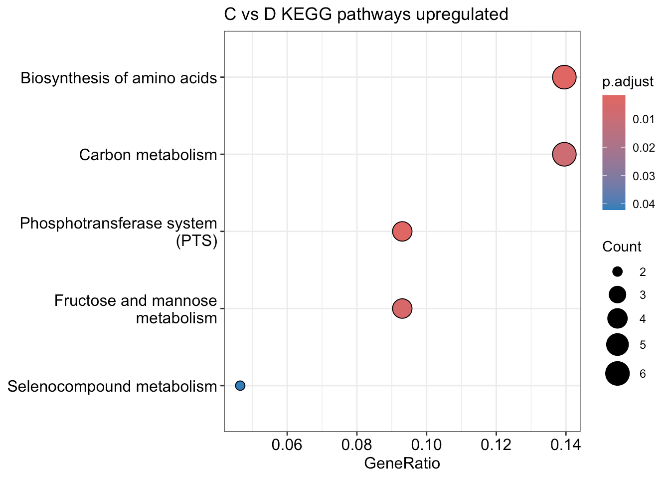

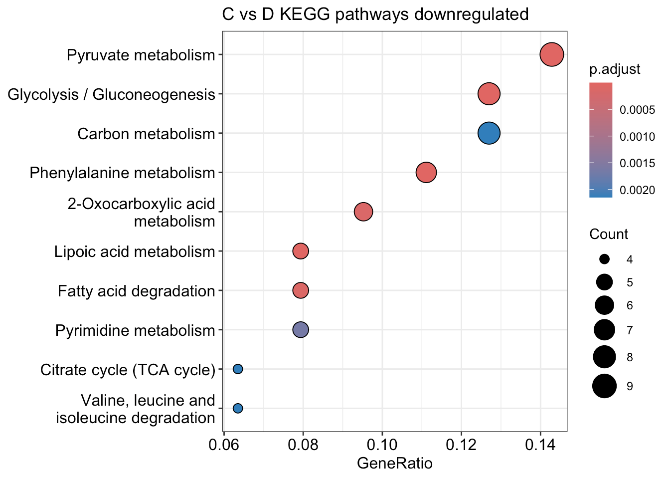


# Figure S17 – Enrichment analysis of **strain D** – KEGG pathways up/downregulated in the RNAseq data. Each panel shows pathways differentially expressed in strain D in the comparison given in the subtitle. For instance, the B vs C upregulated panel shows pathways upregulated in strain D when next to C compared with when next to B.


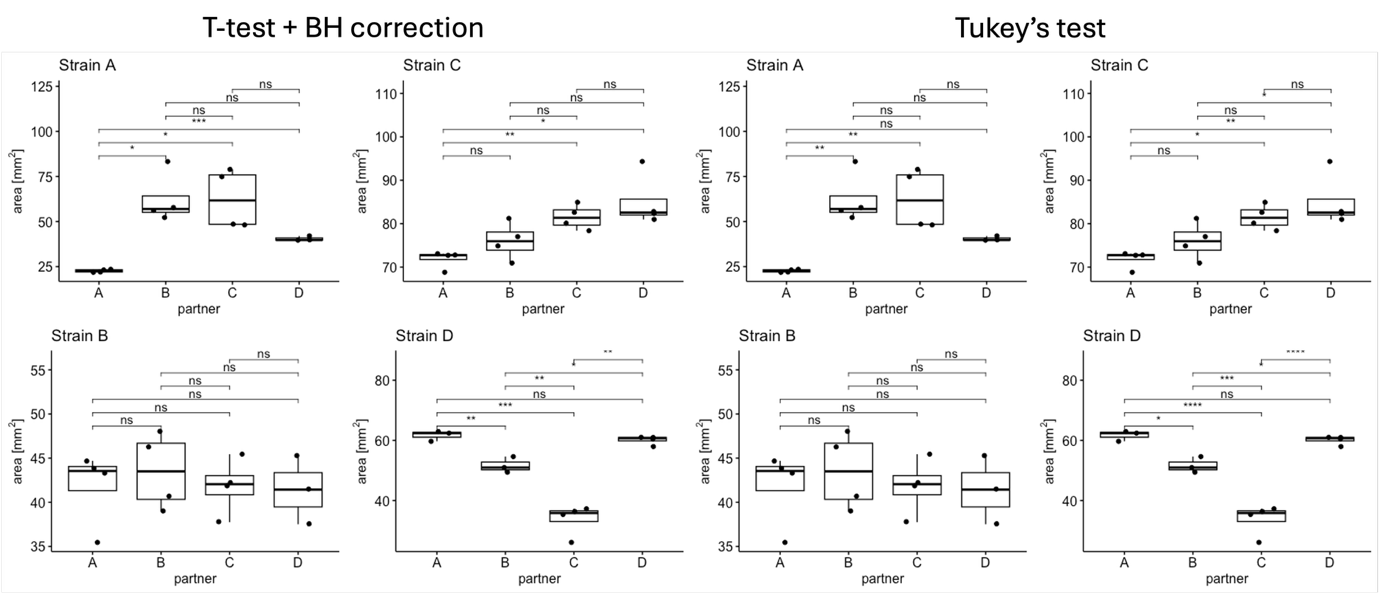


# Figure S18 – Comparison of multiple testing correction methods for pairwise comparisons of computed strain areas (comparative to Figure 2B). Areas were determined algorithmically on day 6, n=4. The manuscript uses multiple t-tests with a Benjamini-Hochberg correction for multiple testing (left panel). Tukey’s Honest Significant Difference test (right panel) does not change the biological interpretation of the significant interactions. Significance levels are indicated as follows: **** = P <0.0001 *** = *P* < 0.001, ** = *P* < 0.01, * = *P* < 0.05

# Supplementary Material References

1. Alanjary, M., Steinke, K. & Ziemert, N. AutoMLST: an automated web server for generating multi-locus species trees highlighting natural product potential. *Nucleic Acids Research* **47**, W276–W282 (2019).

2. Schlatter, D. C., DavelosBaines, A. L., Xiao, K. & Kinkel, L. L. Resource use of soilborne *Streptomyces* varies with location, phylogeny, and nitrogen amendment. *Microbial Ecology* **66**, 961–971 (2013).

3. Flett, F., Mersinias, V. & Smith, C. P. High efficiency intergeneric conjugal transfer of plasmid DNA from *Escherichia coli* to methyl DNA-restricting streptomycetes. *FEMS Microbiology Letters* **155**, 223–229 (1997).

4. Tong, Y. *et al.* Highly efficient DSB-free base editing for streptomycetes with CRISPR-BEST. *PNAS* **116**, 20366–20375 (2019).
